# Phosphate availability and ectomycorrhizal symbiosis with *Pinus sylvestris* have independent effects on the *Paxillus involutus* transcriptome

**DOI:** 10.1007/s00572-020-01001-6

**Published:** 2020-11-16

**Authors:** Christina Paparokidou, Jonathan R. Leake, David J. Beerling, Stephen A. Rolfe

**Affiliations:** Department of Animal and Plant Sciences, University of Sheffield, Sheffield, UK

**Keywords:** *Paxillus involutus*, *Pinus sylvestris*, Ectomycorrhizal symbiosis, Pi-starvation, Pi transporters, RNA-seq

## Abstract

Many plant species form symbioses with ectomycorrhizal fungi, which help them forage for limiting nutrients in the soil such as inorganic phosphate (Pi). The transcriptional responses to symbiosis and nutrient-limiting conditions in ectomycorrhizal fungal hyphae, however, are largely unknown. An artificial system was developed to study ectomycorrhizal basidiomycete *Paxillus involutus* growth in symbiosis with its host tree *Pinus sylvestris* at different Pi concentrations. RNA-seq analysis was performed on *P. involutus* hyphae growing under Pi-limiting conditions, either in symbiosis or alone. We show that Pi starvation and ectomycorrhizal symbiosis have an independent effect on the *P. involutus* transcriptome. Notably, low Pi availability induces expression of newly identified putative high-affinity Pi transporter genes, while reducing the expression of putative organic acid transporters. Additionally, low Pi availability induces a close transcriptional interplay between P and N metabolism. GTP-related signalling was found to have a positive effect in the maintenance of ectomycorrhizal symbiosis, whereas multiple putative cytochrome P450 genes were found to be downregulated, unlike arbuscular mycorrhizal fungi. We provide the first evidence of global transcriptional changes induced by low Pi availability and ectomycorrhizal symbiosis in the hyphae of *P. involutus*, revealing both similarities and differences with better-characterized arbuscular mycorrhizal fungi.

**Supplementary Information:**

The online version contains supplementary material available at 10.1007/s00572-020-01001-6.

## Introduction

The vast majority of plants, including forest trees, form symbioses with mycorrhizal fungi where over 90% of plant roots are primarily connected with the mycelia of ectomycorrhizal (EM) or arbuscular mycorrhizal (AM) fungi (Bonfante and Genre 2010). In the case of EM symbioses, the extensive extraradical mycelium that proliferates through the soil enables forest trees to forage efficiently and acquire nutrients, particularly N and P (Leake et al. 2004). In natural soils, P concentrations in the form of inorganic phosphate (Pi) are often low ranging from 1 to 10 μM (Bieleski 1973). P can be present as organic and inorganic forms, many of which are not directly accessible to plant roots (Costa et al. 2016). The EM fungal mycelium has specialized functions to mobilize, acquire, metabolize, store and transport P from these diverse sources (Nehls and Plassard 2018).

EM fungi contain specialized high-affinity Pi transporters, belonging to the PHT1 family of Pi transporters, that enable the acquisition of Pi present at low concentrations in the soil (Casieri et al. 2013). In the Basidiomycete EM fungus *Tricholoma* spp., Kothe et al. (2002) identified two genes with homology to high-affinity Pi transporters, whose expression was induced under Pi starvation. Moreover, Tatry et al. (2009) identified and functionally characterized two plasma membrane Pi transporter genes, *HcPT1* and *HcPT2*, from *Hebeloma cylindrosporum*, an EM-forming Basidiomycete. Both transporters were proposed to function as high-affinity Pi transporters coupled with H^+^ symporter activity and were expressed during EM symbiosis. However, under Pi-limiting conditions, only the expression of *HcPT1* was induced, suggesting a specific role of *HcPT1* in response to Pi deprivation, with *HcPT2* having a role in Pi-sufficient conditions (Tatry et al. 2009).

The EM mycelium may also release factors that mobilize P from otherwise inaccessible sources (Zhang et al. 2014). For example, the release of low molecular weight organic acids (LMWOAs) can solubilize P from mineral sources such as calcium phosphates, including apatites (Plassard et al. 2011). Phosphatases can also release Pi from organic sources such as phospholipids, nucleic acids and proteins for subsequent uptake by high-affinity Pi transporters (Cairney 2011).

Once Pi is taken up by the EM mycelium some of it is transferred to the host plant while the majority is stored mainly within the fungal hyphae in the form of polyphosphate (poly-Pi) (Bücking et al. 2012). However, the translocation mechanisms of Pi from the extraradical mycelium towards the host plant remain elusive. In conifers, Pi transfer from EM hyphae to the host tree requires Pi efflux across the fungal plasma membrane into the symbiotic apoplastic space, and subsequent Pi transport across the plasma membrane of root epidermal and, to a lesser extent, cortical cells (Smith et al. 1994). According to radiolabelling studies using ^32^P isotopes, the Hartig net is the symbiotic plant-fungus interphase, where Pi transfer takes place (Bücking and Heyser 2003). However, further efforts to elucidate the Pi transfer mechanism across the EM symbiotic interface have not been informative, in contrast to the research carried out in AM symbiosis.

The last step in Pi transport at the fungus-root interphase may be mediated via plant Pi transporters localized in the EM symbiotic apoplastic space, as has been shown in AM symbioses (Parniske 2008). For example, the Pi transporter MtPT4 of *Medicago truncatula* mediates the uptake of Pi via the periarbuscular membrane (Harrison et al. 2002; Javot et al. 2007). Interestingly, MtPT4 is also essential for the maintenance of arbuscules, the symbiotic structure of AM nutrient transport (Javot et al. 2007), suggesting that Pi metabolism and AM symbiosis are intimately connected. In the case of the EM symbiosis, it has been shown that the Pi transporters PtPT9 and PtPT12 from *Populus trichocarpa* were upregulated under Pi starvation (Loth-Pereda et al. 2011).

Studies on the transcriptional response to Pi availability of mycorrhizal fungi while in symbiosis with their host plant are scarce and focus mostly on a few candidate genes. Examples include analyses of expression of fungal Pi transporter genes in response to Pi availability during AM symbiosis. Maldonado-Mendoza et al. (2001) studied the expression and regulation of the AM fungal phosphate transporter gene *GiPT* in the symbiosis between *Medicago truncatula* and *Glomus intraradices*. *GiPT* expression is regulated in response to Pi concentrations surrounding the extraradical mycelium, with *GiPT* transcript levels increasing dramatically at low Pi (35 μM) but decreasing at high Pi (3.5 mM) concentrations. Pi transporter gene expression has also been analysed in the hyphae of the EM fungus *Hebeloma cylindrosporum* in response to Pi availability in symbiosis with *Pinus pinaster* (Tatry et al. 2009). The expression of *HcPT1* is upregulated at low Pi concentrations, suggesting its importance in soil Pi uptake in Pi-limited conditions. In contrast, *HcPT2* is upregulated in sufficient Pi conditions.

The *P. involutus* genome has been sequenced (Kohler et al. 2015) and is available at the MycoCosm genomics resource accessed via the JGI portal (Grigoriev et al. 2014). Casieri et al. (2013) identified three putative Pi transporter genes in the *P. involutus* genome via *in-silico* analysis. However, no analyses of putative *P. involutus* Pi transporter gene expression have been performed. While global transcriptome analyses have been carried out in *P. involutus* using cDNA microarrays, none of them examined specifically the responses of the *P. involutus* mycelium to phosphate availability when in symbiosis with a host plant. For instance, Le Quéré et al. (2005) studied global gene regulation associated with the development of EM symbiosis between *P. involutus* and *Betula pendula*. In addition, Wright et al. (2005) studied the spatial patterns of global gene expression in the extraradical mycelium of *P. involutus* and the mycorrhizal root tips of *B. pendula* while in symbiosis. Moreover, a global gene expression study of *P. involutus* mycelium in response to protein degradation and N assimilation was reported in Shah et al. (2013).

In this study, we explored the role of EM symbiosis and its effect on transcriptional changes in *P. involutus* hyphae under Pi-limiting conditions. We hypothesised that the expression of genes involved in Pi-acquisition would be upregulated under Pi-limiting conditions in a similar manner to AM fungi, but that there would also be other Pi and symbiosis related genes that are unique to the EM interaction. We identified additional putative *P. involutus* Pi transporter genes and characterized their expression in response to varying external Pi concentration and EM symbiosis with *Pinus sylvestris*. Moreover, we unravelled the global changes in gene expression of free-living or symbiotic *P. involutus* mycelium in response to low Pi (0.37 μM) or high Pi (367 μM) concentrations, revealing both similarities and differences with better-characterized AM fungi.

## Materials and methods

### Media

Modified Melin–Norkrans media (MMN medium) was used prepared according to (Müller et al. 2013) including the addition of activated charcoal with Pi, added as KH_2_PO_4_, varying from 0.37 to 3670 μΜ (Supporting Table S1). Media were autoclaved at 15 psi for 15 min at 121 °C and poured into either 90 mm round Petri dishes for non-symbiotic (NS) growth or 100 mm square Petri dishes for symbiotic (S) growth. The surface of the medium was covered with a sterile cellophane membrane, which was rinsed with ultrapure water to remove any potential external phosphate contamination.

### Fungal material

For NS growth, *P. involutus ATCC 200175* was grown on 10% MMN fungal culture media (Supporting Table S2) to provide an inoculum. Plugs of mycelia were cut with a 6-mm diameter cork-borer and used to inoculate MMN-NS medium with different Pi concentrations (Supporting Table S1).

For symbiotic growth, *P. sylvestris* seeds (Forestry Commission, UK) were surface sterilized with 30% (*v*/*v*) H_2_O_2_ and germinated on 1.2% (*w*/*v*) plant agar (Duchefa). Seedlings were transferred to Petri dishes containing MMN-S medium with different Pi concentrations (Supporting Table S1) and inoculated with a 6-mm diameter fungal plug, originating from 10% MMN fungal culture media (Supporting Table S2).

### Determination of fungal and plant dry mass

Fungal hyphae were collected using a surface scraper tool and carefully separated from EM root tips, placed into 2 mL Eppendorf tubes and oven-dried at 90 °C for up to 5 days. Plant material was placed in paper envelopes and dried in the same manner. Dry mass measurements were performed as soon as the tissues were completely dry using an analytical balance (Mettler AT261B Delta Range Balance). Results are the average dry mass of three to six biological replicates.

### Determination of fungal and plant Pi content

Pi content from 30 mg of fresh plant tissue (needles) or fungal hyphae (see above), or 10 mg of fresh plant root tissue, was measured using the Phosphate Assay Kit (Colorimetric) Ab65622 according to the manufacturer’s instructions (Abcam). The assay utilizes a proprietary formulation of malachite green and ammonium molybdate that forms a chromogenic complex with Pi ions, leading to an intense absorption band around 650 nm. The kit detects all types of Pi, such as PO_4_^3−^, HPO_4_^2−^ and H_2_PO_4_^−^. For each sample, the Pi content (nmol) is referenced to a standard curve. Results are the average Pi content of three biological replicates.

### Bioinformatic analysis of *P. involutus* ATCC 200175 Pi

A search was performed on the MycoCosm database (Grigoriev et al. 2014) accessible via the JGI portal (Grigoriev et al. 2012; Nordberg et al. 2014) for Pi transporter genes with the EuKaryotic Orthologous Groups (KOG) tool (Koonin et al. 2004), resulting in the identification of seven putative *PiPTs* (*PiPT1-7*). The genomic sequences of the *PiPTs* were retrieved from the MycoCosm database (Supporting Note S1). The putative amino acid sequences for all the PiPTs were retrieved according to their nucleotide transcript sequence listed on MycoCosm with the ExPASy translate tool (Artimo et al. 2012) (Supporting Note S2). Supporting Table S3 summarizes various characteristics of the *PiPT* genes and predicted proteins including the number of exons, the isoelectric point (pI) and molecular weight (Mw) calculated using the ExPASy compute pI/Mw tool, and predicted subcellular localisation using the WoLF PSORT server (Horton et al. 2007).

### Phylogenetic analysis of PiPTs

A phylogenetic analysis was carried out based on the Neighbour-Joining method using MEGA 7 (Zuckerkandl and Pauling 1965; Saitou and Nei 1987; Kumar et al. 2016). The evolutionary relationship of PiPTs was inferred using the maximum likelihood method based on the Whelan and Goldman model (Whelan and Goldman 2001). The proportion of replicate trees in which the associated taxa clustered together in the bootstrap test (1000 replicates) was calculated (Felsenstein 1985) and branches corresponding to partitions reproduced in less than 50% of the bootstrap replicates collapsed. Initial tree(s) for the heuristic search were obtained automatically by applying Neighbour-Joining and BioNJ algorithms to a matrix of pairwise distances estimated using a JTT model and then selecting the topology with superior log-likelihood value. The tree is drawn to scale with branch lengths measured as the number of substitutions per site. Protein sequence similarity analysis for the putative *P. involutus* PTs PiPT3, PiPT5 and PiPT7 which clustered separately from other characterized PTs was performed using BLAST (sequence identity threshold > 75%, Supporting Note S3).

### RNA extraction and semi-quantitative reverse transcription PCR

Total fungal RNA was extracted with the E.Z.N.A. ® Fungal RNA Kit according to the manufacturer’s protocol from extraradical fungal hyphae. For qRT-PCR analysis, 500 ng of total RNA was used for cDNA first-strand synthesis (SuperScript® III First-Strand Synthesis SuperMix). For qRT-PCR reactions, a Rotor-Gene SYBR Green PCR Kit (Qiagen) together with gene-specific primers were employed in conjunction with a Rotor-Gene Q Real-Time PCR Cycler (Qiagen) according to the manufacturer’s instructions. Gene-specific primers used in qRT-PCR reactions are shown in Supporting Table S4. The reaction conditions (two-step with a melt curve) were 95 °C for 10 min, 35 cycles of 95 °C for 10 s and 60 °C for 55 s. High-resolution melting analysis was carried out to detect primer-dimers or other non-specific amplification products. Relative gene expression was quantified based on a modified Livak’s ΔΔ^CT^ method (Livak and Schmittgen 2001) with correction for the reaction efficiency of each sample (Pfaffl 2001). Relative gene expression was normalized against the average expression values of the housekeeping gene *ACTIN*, which was identified as a suitable control according to the RefFinder tool (Xie et al. 2012). For each qRT-PCR reaction, three biological replicates were analysed per condition per Pi concentration.

### Statistical analyses

All statistical analyses were performed in R (R Core Team 2018). Statistically significant differences in hyphal/plant dry mass, Pi content and relative mRNA expression between different Pi concentrations were determined by analysis of covariance (ANCOVA). To determine if values differed at specific supplied Pi concentrations, an ANOVA was performed followed by Tukey’s post-hoc analysis for multiple comparisons (*α* = 0.05). Multivariate analysis of variance (MANOVA) on distance matrices between conditions of the RNA-seq experiment was performed using the ‘adonis’ function of R package ‘vegan’ (*α* = 0.05) (Oksanen et al. 2019).

### Microscopy and imaging

Images were taken with a Leica stereoscope and SPOT advanced software.

### RNA-seq library preparation and sequencing

RNA-seq libraries were prepared from total RNA samples, extracted from *P. involutus ATCC 200175* hyphae grown at low Pi (0.37 μM Pi) and high Pi (367 μM Pi) in symbiotic and non-symbiotic conditions. Libraries were prepared using a TruSeq stranded mRNA library preparation kit (Illumina) at the University of Edinburgh (Edinburgh Genomics). Three biological replicates were used for low Pi NS and S conditions, and five biological replicates were used for high Pi NS and S conditions, respectively. In total, 26–45.9 M read pairs were obtained per sample (HiSeq 4000 75 bp Paired End).

### RNA-seq data analysis, read trimming, reference genome, read alignment, read counting and count pre-processing

Default values were used for all parameters in all software used unless otherwise stated. Reads were trimmed using Cutadapt version cutadapt-1.9.dev2 (Martin 2011). Reads were trimmed for quality at the 3′ end using a quality threshold of 30 and for adapter sequences of the TruSeq stranded mRNA kit (AGATCGGAAGAGC). Reads after trimming were required to have a minimum length of 50. Between 25.4–44.8 M reads were obtained after trimming from all samples (97–98.7% of the input reads). The reference genome used for mapping was the *P. involutus* genome from Ensembl, assembly *ATCC 200175*, annotation version 1 (accession number GCA_000827475). Reads were aligned to the reference genome using STAR (Dobin et al. 2013) version 2.5.2b specifying paired-end reads and the option ‘-outSAMtype BAM Unsorted.’ In all samples, 24–42.5 M trimmed read pairs aligned to the reference genome (88.3–94.9%). Reads were assigned to exon features grouped by gene id in the reference genome using ‘featureCounts’ (Liao et al. 2013), which assigns counts on a ‘fragment’ basis as opposed to individual reads such that a fragment is counted where one or both of its reads are aligned and associated with the specified features. Strandness was set to ‘reverse,’ and a minimum alignment quality of 10 was specified. Gene names and other fields were derived from the input annotation and added to the count matrix. Among all samples, 18.1–34.8 M read pairs were aligned to exon features for counting (75.7–83.6%). The raw counts table was filtered to remove rows consisting predominantly of near-zero counts, filtering on counts per million (CPM) to avoid artefacts due to library depth. Specifically, a row of the expression matrix was required to have values greater than 0.1 in at least 3 samples.

### Count normalization, exploratory analysis and differential analysis

Read counts were normalized through the R package “DESeq2”, which applies a negative binomial distribution and shrinkage estimation of gene dispersion (Love et al. 2014). Using the “DESeq2” package, a principal component analysis (PCA) on *rlog*-transformed read counts (Love et al. 2014) was performed in order to visualize sample-to-sample differences. Differential gene expression analysis was carried out in DESeq2 using the Wald test, selecting genes with false discovery rate (FDR) corrected *p* value (Q-value, Benjamini-Hochberg correction) < 0.05 and log2 fold change (FC) > 1 for each indicated comparison.

### RNA-seq data visualization

For each pairwise comparison in Table 2, the distribution of genes was visualized through Volcano plots produced using the R packages ‘ggplot2’ (Wickham 2016). The Log2 fold change of 3167 *P. involutus ATCC 200175* genes showed statistically significant differential expression in one or more pairwise comparisons (as listed in Table 2, log2 FC > 1 threshold) and were visualized using the ‘pheatmap’ package for R (Kolde 2015).

### GO terms enrichment analysis

For each selection of genes indicated, significant enrichment of GO terms from the molecular function (MF) aspects was calculated with the “GOstats” package for R (Falcon and Gentleman 2006), using a custom Blast2GO annotation as background (Hypergeometric test, *p* < 0.05). Predicted gene sequences from the *P. involutus* genome annotation were categorized into GO categories using Blast2GO (Conesa et al. 2005; Conesa and Götz 2008; Götz et al. 2008).

### Data availability

Aligned sequences data (BAM files) from the transcriptome sequencing in this study have been deposited in the European Nucleotide Archive (ENA) at EMBL-EBI under accession number PRJEB35619.

## Results

### Responses of *P. involutus* to Pi

**Fig. 1:**
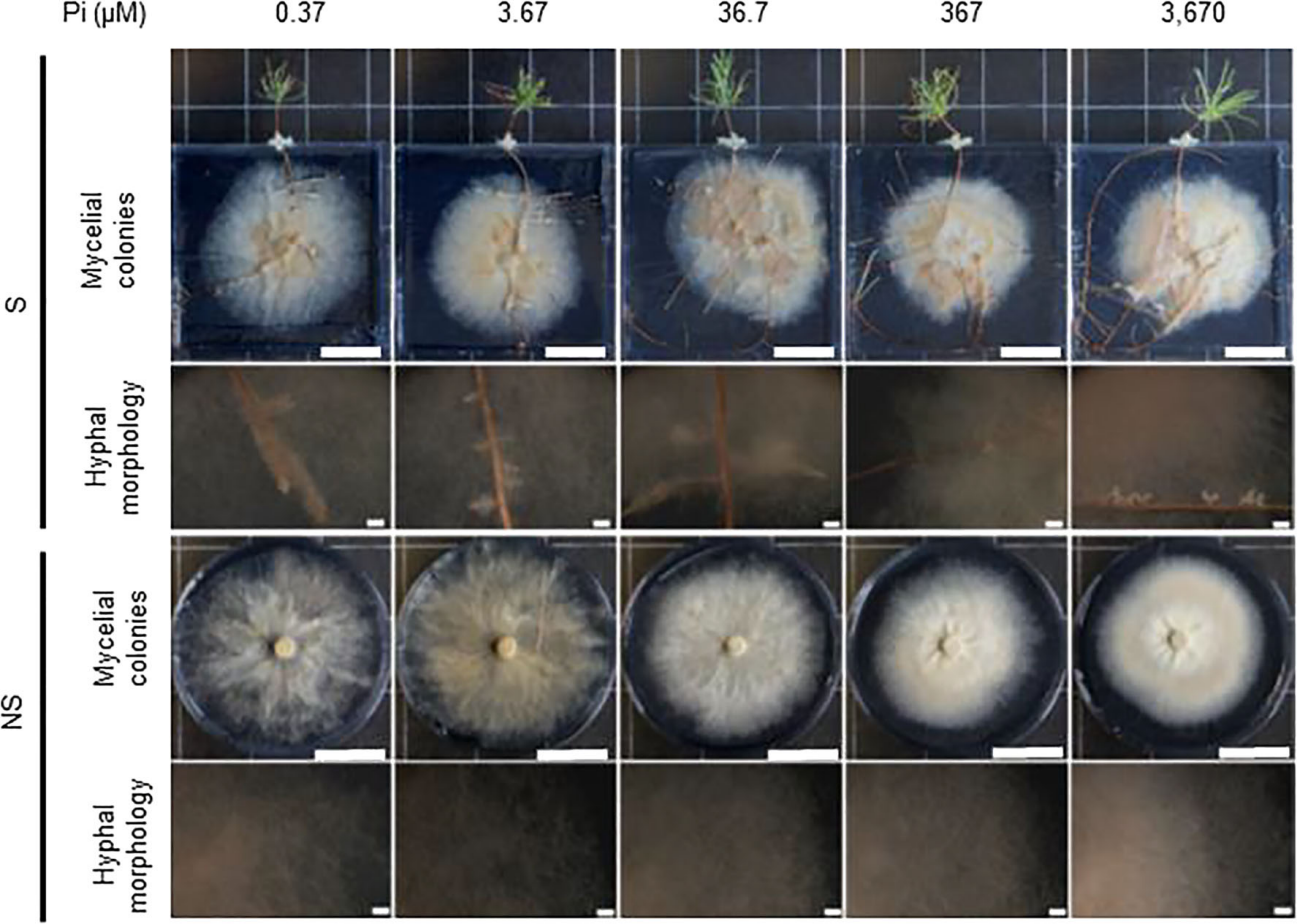
Growth of *P. involutus ATCC 200175* after 90 days on MMN medium containing different concentrations of Pi. Representative images of mycelium in EM symbiosis with *P. sylvestris* seedling (S) or non-symbiotically (NS) are shown. Scale bars 3 cm. For each condition, the lower panel shows detail of hyphal morphology. Scale bars 1 mm

**Fig. 2:**
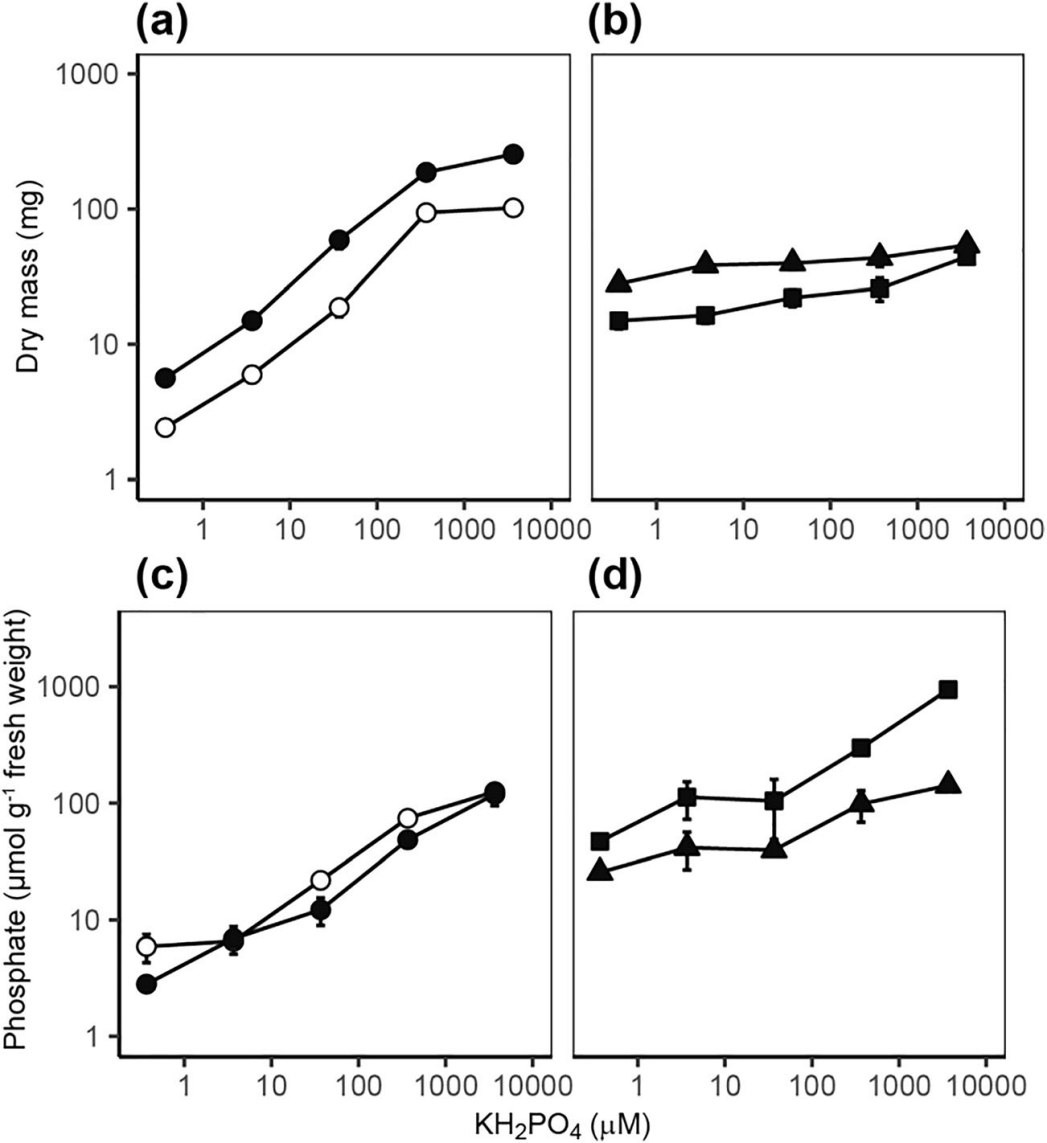
Dry mass and phosphate content of *P. involutus ATCC 200175* fungal and *Pinus sylvestris* plant material. (a) Dry mass of hyphae for NS (open symbols) and S (closed symbols) conditions. (b) Dry mass of *P. sylvestris* needles (triangles) and roots (squares) in S condition. (c) Pi content of hyphae for both NS and S conditions. (d) Pi content of needles and roots in the S condition. Results are the mean ± standard errors of three to six biological replicates

In order to select the appropriate low and high Pi concentrations for global-scale gene expression analyses using RNA-seq, *P. involutus ATCC 200175* was grown on either symbiotic (S) or non-symbiotic (NS) MMN media with supplied Pi concentrations ranging from 0.37 to 3670 μM. Pictures of the mycelium are shown in Fig. 1 and the analysis of mycelial and plant dry mass and P content shown in Fig. 2. At the lowest Pi concentration (0.37 μM), the mycelium grew rapidly but with a disperse hyphal morphology in both S and NS conditions. At higher Pi concentrations the mycelium became increasingly compact. The dry mass of the mycelium increased with increasing Pi supply in both NS and S conditions and the mycelial mass was greater in the NS condition compared to the S condition (ANCOVA, *p* = 0.006) but there was no significant interaction between Pi supply and symbiotic status (*p* = 0.833) (Fig. 2a). The dry mass of plant roots and needles also increased with Pi supply (Fig. 2b). The Pi content of *P. involutus* hyphae also increased with increased Pi supply (Fig. 2c) with slightly greater values in the NS condition compared to the S condition (ANCOVA, *p* = 0.038) but again, there was no significant interaction between Pi supply and symbiotic status (*p* = 0.101). The needle and root Pi content of seedlings also increased with increasing Pi supply (Fig. 2d). Notably, increasing Pi supply had no significant effect on *P. involutus ATCC 200175* mycorrhization rates of *P. sylvestris* roots (Fig. S1; one-way ANOVA, *p* > 0.05).

### Identification of *P. involutus* ATCC 200175 putative Pi transporter genes, phylogenetic and expression analysis

Seven putative *PiPT* genes were identified within the *P. involutus ATCC 200175* genome (Supporting Table S3) that encode Pi transporter proteins (PiPTs). PiPT1-7 belongs to the major facilitator superfamily (MFS) of transporters. The MFS family of transporters includes single-polypeptide secondary carriers capable of ion and small solute transport across cell membranes. MFS transporters generally depend on chemiosmotic gradients across cell membranes and are described as secondary rather than primary transporters activated by ATP hydrolysis (Pao et al. 1998). External Pi uptake has been traditionally attributed to the MFS transporters such as the Pht1 family of plant Pi transporters. Plant Pht1 transporters have been identified by their similarity to the *Saccharomyces cerevisiae* high-affinity Pi transporter, ScPho84, which mediates external Pi uptake across the plasma membrane via H^+^ symport activity (Remy et al. 2012). Similarly, in EM fungi such as *Hebeloma cylindrosporum*, high-affinity Pi transporters were identified and functionally characterized, demonstrating a high similarity to ScPho84 and exhibiting Pi: H^+^ symporter activity (Tatry et al. 2009).

**Fig. 3:**
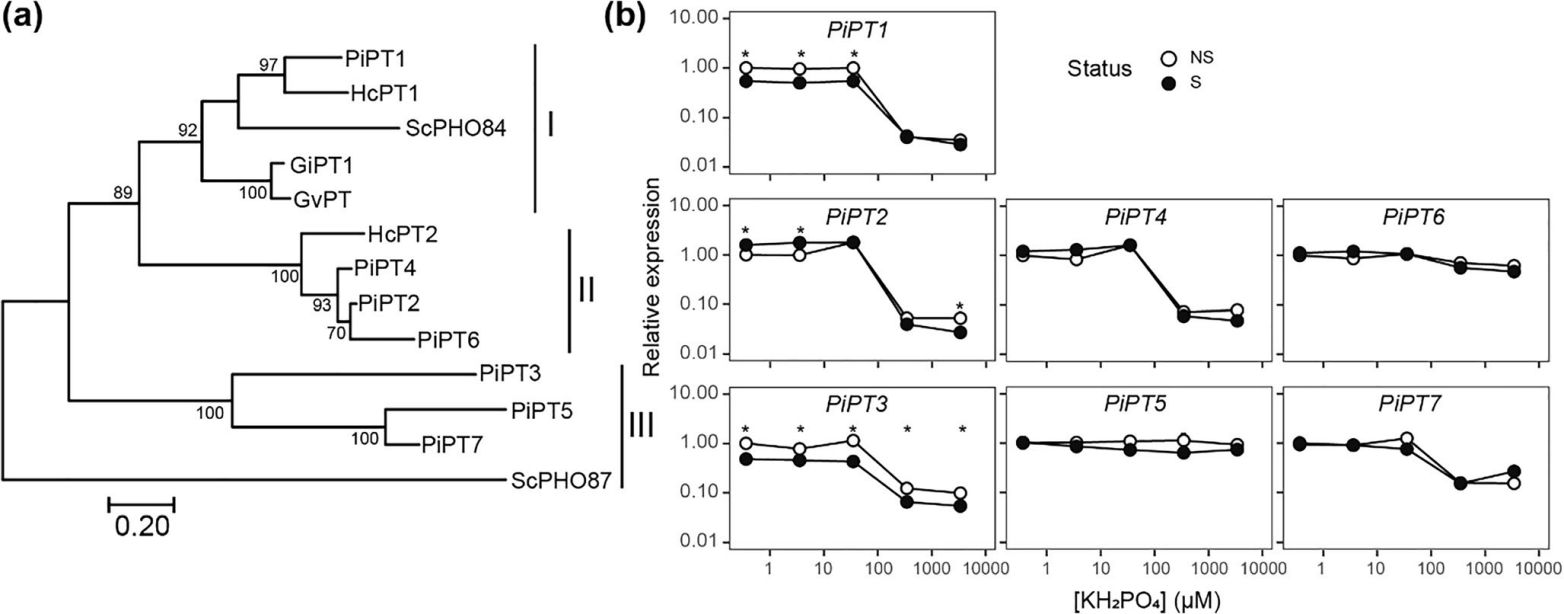
Phylogenetic and expression analysis of putative *P. involutus ATCC 200175* phosphate transporters. (a) A phylogenetic analysis of PiPTs and selected other known phosphate transporters using the maximum likelihood method based on the Whelan and Goldman model. The proportion of replicate trees in which the associated taxa clustered together in the bootstrap test are shown next to the branches. The transporters clustered into 3 groups. (b) Relative *mRNA* expression of the *PiPTs* for NS (open circles) and S (closed circles) growth conditions measured after the growth of mycelium for 90 days with different Pi supplies. Results are represented as fold-change relative to the expression at 0.367 μM in the NS condition and are the mean ± standard error of 3 biological replicates. Asterisks indicate that expression differed significantly between NS and S conditions

A phylogenetic tree of the seven putative PiPTs was constructed (Fig. 3a), together with previously characterized high-affinity Pi transporters of *Glomus intraradices* (GiPT) (Maldonado-Mendoza et al. 2001), *Glomus versiforme* (GvPT) (Harrison and Buuren 1995), *S. cerevisiae* (ScPHO84) (Bun-Ya et al. 1991), *Hebeloma cylindrosporum* (HcPT1 & HcPT2) (Tatry et al. 2009) and the low-affinity Pi transporter of *S. cerevisiae*, PHO87 (Pinson et al. 2004; Hürlimann et al. 2009). PiPT1 clustered with the high-affinity Pi transporters HcPT1 and ScPHO84. PiPT2, PiPT4 and PiPT6 grouped with the high-affinity Pi transporter HcPT2. In contrast, PiPT3, PiPT5 and PiPT7 did not cluster with any of the high-affinity Pi transporters used in the phylogenetic analysis, nor the low-affinity Pi transporter ScPHO87.

The expression of *PiPT1-PiPT7* in *P. involutus* hyphae was determined using qRT-PCR in NS and S conditions (Fig. 3b). The majority of the *PiPTs* genes were responsive to Pi-concentration in both the NS and S conditions. In particular, *PiPT1*, *PiPT2*, *PiPT3*, *PiPT4* and *PiPT7* transcripts showed higher expression at low Pi supply (up to 36.7 μM) compared to high Pi availability (367 μM and above). In contrast, the expression of *PiPT5* and *PiPT6* was not strongly affected by Pi availability, but the expression of these transporters was low making precise quantification difficult. The expression of most *PiPT3* was significantly greater in the NS condition compared to the S condition at all concentrations (ANCOVA *p* = 0.0019). This was also true for *PiPT1,* but only at the lower Pi supply rates (36.7 μM and below). In contrast, the expression of *PiPT2* was slightly higher in the S condition than the NS condition at 0.367 and 3.67 μM Pi concentrations.

### Global patterns of gene expression

**Fig. 4:**
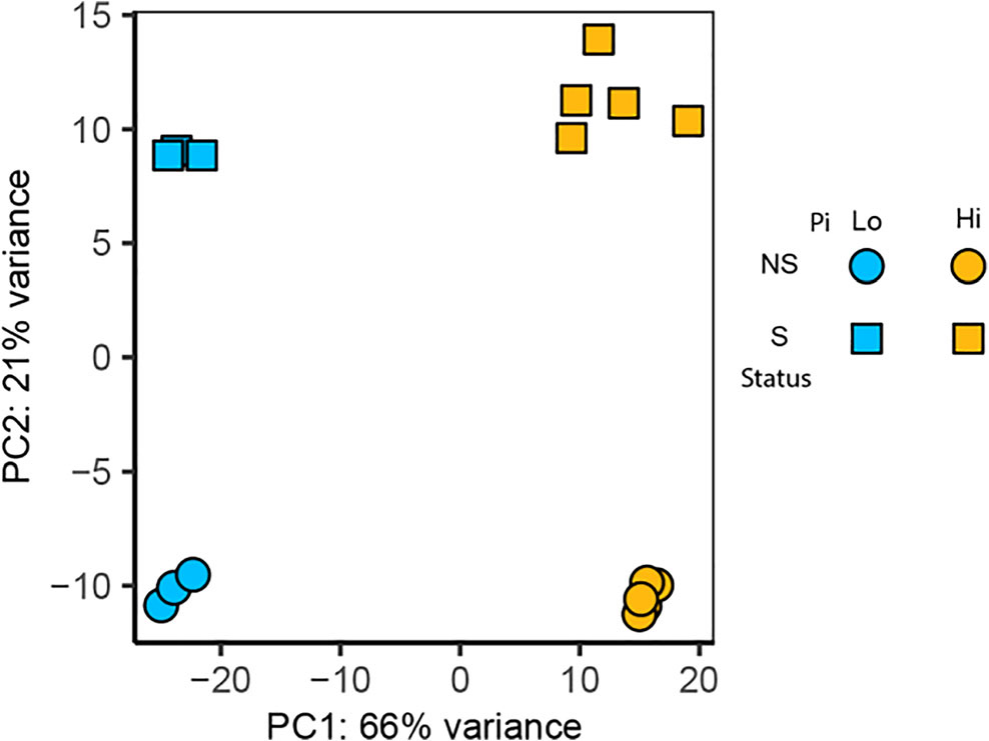
Principal component analysis of RNA-seq samples. Mycelium was collected from *P. involutus ATCC 200175* after 90 days of growth on media with high (367 μM) or low (0.367 μM) Pi availability grown non-symbiotically (NS) or symbiotically (S) with *P. sylvestris* seedlings

RNA-seq analysis was performed on total RNA extracted from *P. involutus ATCC 200175* hyphae grown on MMN at low Pi (0.37 μm) and high Pi (367 μm) in both NS and S conditions. A Principal Components Analysis of *rlog*-transformed read counts showed clear separation of the experimental conditions (Fig. 4). Principal component 1 (PC1) showed separation based on the Pi supply, whilst PC2 showed separation on the symbiotic status (NS vs S). Together, PC1 and PC2 explained 88% of the total variance of the dataset (Fig. 4). However, PERMANOVA did not identify any significant interaction between the supplied Pi concentration and the symbiotic status within the experimental system (Table 1).

**Table 1:** Nonparametric multivariate analysis of variance between conditions of the RNA-seq experiment

Conditions compared	*F* model	*R*^2^	Pr value (> *F*)
Symbiosis	11.2343	0.20945	0.001 ***
Pi supply	28.0350	0.52267	0.001 ***
Symbiosis: Pi supply^1^	2.3687	0.04416	0.089

^1^Interaction effect

### Differential gene expression analysis

**Fig. 5:**
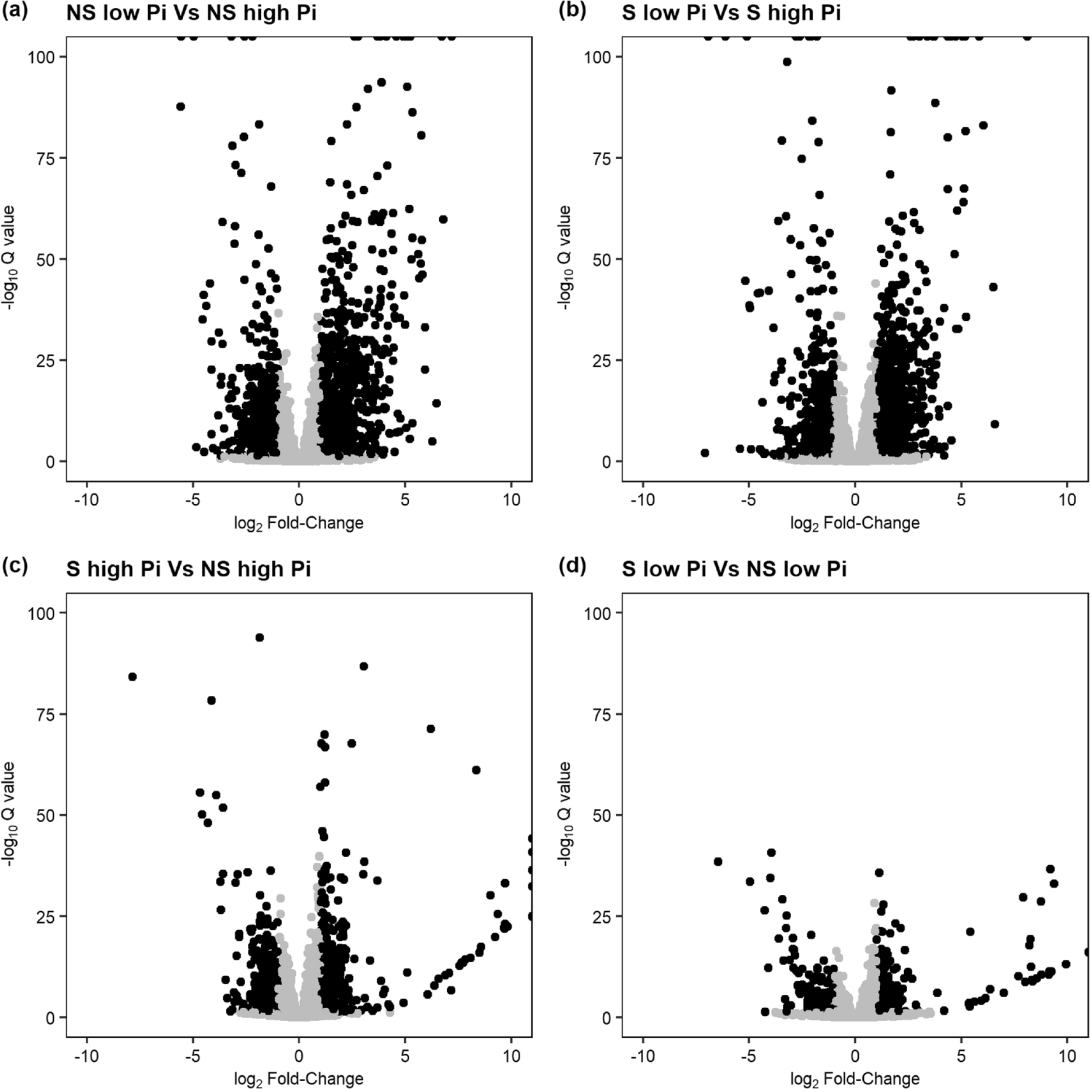
Volcano plots showing differential gene expression resulting from Pi supply or symbiosis status. NS: *P. involutus ATCC 200175* hyphae grown in non-symbiotic conditions; S: *P. involutus ATCC 200175* hyphae grown in EM symbiosis with *P. sylvestris* seedlings; Low Pi: 0.37 μM Pi; High Pi: 367 μM Pi. Black dots indicate genes (see Table 1) showing statistically significant differential expression (Q-value <0.05) with an absolute log_2_ fold change > 1. –log10 Q values that exceeded 100 or log_2_ fold-changes that exceed 10 are shown at the axis limits

Differential gene expression analysis of the RNA-seq dataset was carried using a generalized linear model implemented in DESeq2 with pairwise comparisons between each condition. Comparisons between low Pi and high Pi, either in NS or S conditions, identified the greatest numbers of up- or downregulated genes, indicating that the majority of the differentially expressed genes were regulated by Pi supply (Table 2, comparisons 1 and 2). Comparisons between NS and S conditions, either in low Pi or high Pi, identified fewer up- or downregulated genes. Nonetheless, a large number of genes were identified to be up- or downregulated by symbiosis in both high and low Pi supply (Table 2, comparisons 3 and 4). The distribution of differentially expressed genes showing statistically significant up- or downregulation in each pairwise comparison was visualized through volcano plots (Fig. 5).

**Table 2:** Number of genes showing statistically significant up- or downregulation in each pairwise comparison between conditions of the RNA-seq experiment

Conditions compared	Upregulated	Downregulated
NS low Pi Vs NS high Pi	1173	772
S low Pi Vs S high Pi	1197	824
S high Pi Vs NS high Pi	446	383
S low Pi Vs NS low Pi	236	193

*NS*, non-symbiotic condition; *S*, symbiotic condition; *low Pi*, 0.37 μM Pi; *high Pi*, 367 μM Pi

### Clustering of gene expression

**Fig. 6:**
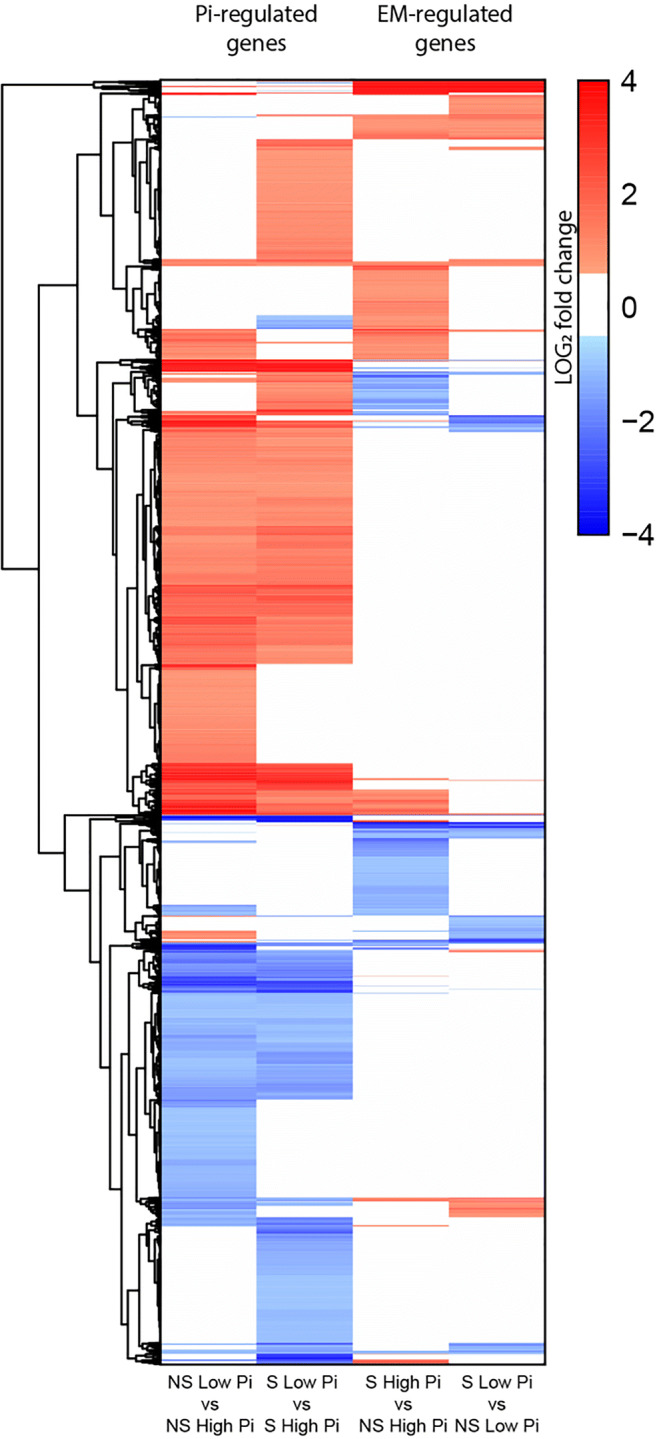
Heatmap clustering of 3167 of differentially regulated genes. *P. involutus ATCC 200175* genes with statistically significant differential expression (Wald test, Q-value < 0.05) in one or more pairwise comparisons (Table 1). Colours are log2-transformed fold changes (values above 4 are shown as 4 for clarity)

A total of 3167 unique *P. involutus* genes showed a statistically significant differential expression in one or more pairwise comparisons (Wald test, *Q* value < 0.05). The log2 fold changes are shown as a heatmap, clustered according to expression (Fig. 6, Supporting Table S5). The largest clusters of expression profiles were genes up- or downregulated by low Pi in either, or both, the NS or S condition. Fewer genes were up- or downregulated specifically by EM symbiosis, either at low or high Pi or both. For a small proportion of genes, up- or downregulation by low Pi supply was further enhanced or repressed by the EM symbiosis. All PiPTs were upregulated by low Pi in both NS and S conditions and, overall, the patterns of expression were comparable to those seen with qRTPCR (Fig. S2). The expressions of PiPT5 and PiPT6 were much lower than that of the other PiPT genes but consistently (and statistically significantly) higher in low Pi conditions compared to high Pi conditions.

**Fig. 7:**
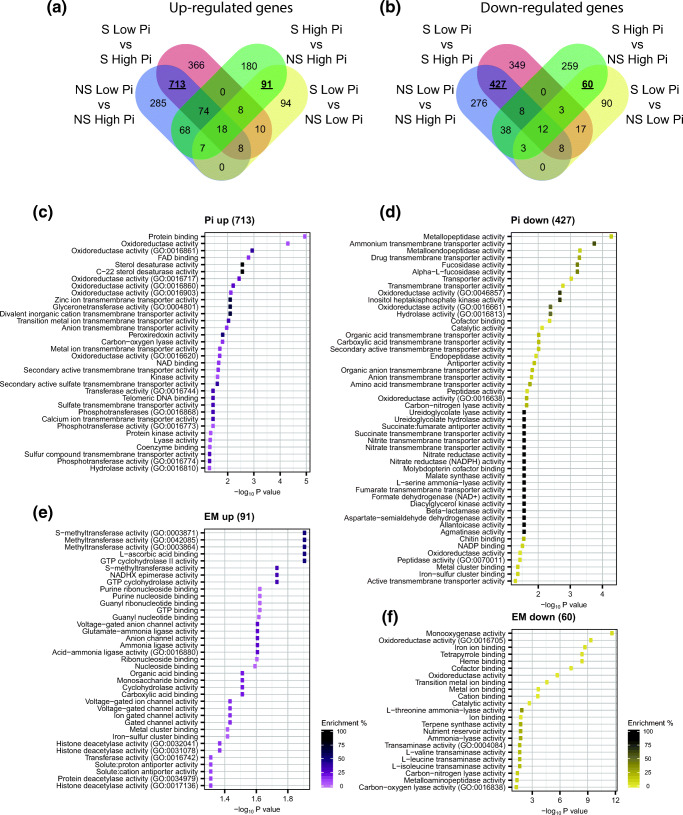
GO analysis of Pi- and EM-regulated genes in *P. involutus*. Venn diagrams showing the relationships between *P. involutus ATCC 200175* genes that were significantly (a) up- or (b) downregulated in each pairwise comparison. Molecular function (MF) GO terms showing significant enrichment (*P* value < 0.05) in selections of up- (c) or downregulated (d) genes by Pi in both NS and S conditions or up- (e) or downregulated (f) genes by EM symbiosis in both low Pi and high Pi conditions. Colours indicate the enrichment percentage of each GO term listed

Four-way Venn diagrams were used to identify the number of genes showing statistically significant up- or downregulation in one or more of the four pairwise comparisons described in Table 2 (Fig. 7). Seven hundred thirteen (713) genes were upregulated (Fig. 7a), and 427 were downregulated by low Pi in both NS and S conditions (Fig. 7b). Similarly, 91 genes were found to be upregulated (Fig. 7a) and 60 genes downregulated by the presence of EM symbiosis in both high and low Pi conditions (Fig. 7b, Supporting Table S6).

### GO term analysis

The 713 genes upregulated by low Pi showed statistically-significant enrichment in GO terms related to phosphate mobilization, primary metabolism and ion transport (Fig. 7c). On the other hand, the 427 genes downregulated by low Pi showed statistically significant enrichment in GO terms related to the transport of larger molecules such as organic acids, as well of GO terms related to nitrogen metabolism, transport and acquisition (Fig. 7d). The 91 genes upregulated by EM symbiosis showed significant enrichment in GO terms related to ion transport, GTP and amino acid syntheses, such as terms related to L-glutamine metabolism or tetrahydrofolate synthesis (Fig. 7e). The 60 genes downregulated by EM symbiosis were significantly enriched in GO terms related to monooxygenase activity, metal ions binding and tetrapyrrole cofactors binding (Fig. 7f). Indeed, nine out of 60 genes in this selection are annotated as putative cytochromes P450 (CYPs) (Supporting Table S6), widespread heme-containing monooxygenases with diverse functions in primary and secondary metabolism, as well as xenobiotic compounds degradation (Shin et al. 2018). Comparison with functionally classified CYPs from different fungal species (Moktali et al. 2012), showed that these genes are more closely related to CYPs with roles in secondary metabolism and, to a lesser extent, in xenobiotic compounds detoxification (Fig. S3).

## Discussion

### Pi availability and EM symbiosis trigger global gene expression changes in *P. involutus* independently from one another

The symbiotic partnership of EM fungi with trees facilitates the acquisition of limiting nutrients such as P (Leake et al. 2004). Analysis of global transcription patterns provides a comprehensive view of expression changes that occur in this symbiosis but previous studies have focused on the mycorrhization process, AM mycorrhiza and/or changes in plant gene expression only (Le Quéré et al. 2005; Wright et al. 2005; Shah et al. 2013; Calabrese et al. 2017; Vangelisti et al. 2018; An et al. 2018). Studies that have examined the transcriptional response of mycorrhizal fungi to Pi availability have mostly focused on a few candidate genes (Maldonado-Mendoza et al. 2001; Tatry et al. 2009). We utilized an artificial system which replicates the low Pi availability of natural soils (Fig. 1), and used it to explore global transcriptional changes in the hyphae of EM fungus *P. involutus*, growing either in symbiosis with its host tree *P. sylvestris* or alone. This study provides the first evidence of the effect of both Pi availability and EM symbiosis on global gene expression patterns in *P. involutus*. Notably, both exploratory analysis and differential gene expression analysis confirmed that low Pi availability has a larger impact on *P. involutus* transcriptome compared to presence or absence of EM symbiosis (Figs. 4, 5 and 6 and Table 2). Our global transcriptional analysis was performed on hyphae derived from extraradical mycelium only, highlighting the systemic changes induced by EM symbiosis in the *P. involutus* mycelium. Specific analysis of EM root tips may yield different, localized transcriptional changes induced by EM symbiosis. Our results indicate that Pi availability and EM symbiosis affect global changes in *P. involutus* gene expression independently from one another (Table 1). Indeed, by separating differentially expressed genes based on their specific expression profiles, only 26 upregulated and 20 downregulated genes appear to be co-regulated by the presence of EM symbiosis and low Pi availability, and their putative functions are mostly unknown (Fig. 7a,b; Supporting Table S6).

### *P. involutus* transport genes are pivotal for the response to low Pi availability

Multiple studies have indicated the importance of high-affinity transporters in Pi foraging and acquisition by mycorrhizal fungi (Maldonado-Mendoza et al. 2001; Tatry et al. 2009; Sun et al. 2019). In this study, we identified seven putative high-affinity Pi transporters in *P. involutus* genome, which were all found to be strongly upregulated in low Pi availability conditions from either qPCR or RNA-seq gene expression analysis (Fig. 2 and Supporting Table S5). Functional characterization of 713 genes specifically upregulated by low Pi availability also revealed a strong enrichment for GO terms related to ion transport (Fig. 7c), confirming the importance of high-affinity transporters in the uptake of limiting nutrients such as P. Among genes differentially regulated by Pi availability, many were found to encode putative synaptic vesicle transporters belonging to the major facilitator superfamily (MFS) of transporters (Supporting Table S5), providing a first indication that vesicle-mediated transport may play a direct role in Pi transport and mobilization in EM.

The EM fungus *P. involutus* is capable of dissolution Pi-rich minerals such as apatite, providing a source of Pi for itself and its host plant (Smits et al. 2012). The dissolution process involves the production and excretion of organic acids onto the mineral surface, which can be enhanced by the photosynthates provided by the host plant during EM symbiosis (Drever and Vance 1994; Schmalenberger et al. 2015). Interestingly, GO term analysis of the 427 genes specifically downregulated by low Pi availability identified multiple terms related to organic acid transport (Fig. 7d). The suppression of organic acid transport in the absence of a dissoluble P source, albeit in limiting Pi conditions, provides an interesting clue to the regulation of *P. involutus* in dissoluting activity, since organic acid transport and metabolism genes were found to be upregulated in limiting Pi conditions but in presence of hydroxyapatite (Paparokidou et al. unpublished results). Together, these results suggest that *P. involutus* is able to recognize the presence or absence of a Pi-rich dissoluble source and adjust its transcriptional response accordingly.

### *P. involutus* genes differentially regulated by low Pi availability and EM symbiosis: similarities and differences with AM fungi

Interestingly, low Pi availability negatively regulates primary metabolism in *P. involutus* hyphae, in particular, genes significantly enriched for functional GO terms related to N metabolism, acquisition and transport (Fig. 7d). This finding suggests a close interplay between the metabolism of essential elements P and N by the fungal hyphae at the transcriptional level, indicating that when P availability is low, N-related metabolic genes are downregulated. Similarly, accumulation and assimilation of ammonium were increased in *Trifolium subterraneum L.* and *Allium cepa* plants during mycorrhizal symbiosis with the AM fungus *Glomus mosseae*, following relief from Pi starvation stress (Smith et al. 1985). Notably, transcriptomic analysis of hyphae from the AM fungus *Rhizophagus irregularis* colonizing *Lotus japonicus* roots at varying levels of Pi showed a reduction in expression of cell cycle-related genes at lower Pi concentrations, but no alterations in primary metabolism or transport genes, as reported in this study (Sugimura and Saito 2016). It must be noted, however, that the RNA-seq experiment presented in Sugimura and Saito 2016 study showed very low coverage, which could prevent adequate quantification of many lowly-expressed genes (Conesa et al. 2016).

Among the genes specifically upregulated by EM symbiosis with *P. sylvestris,* significant enrichment was found for GO terms related to GTP synthesis and signalling (Fig. 7e). These results indicate that GTP signalling may play a role in the maintenance of EM symbiosis. This hypothesis is partially supported by a previous transcriptomic study, which identified significant enrichment of GO terms related to GTP signalling during the initial stages of AM symbiosis between the AM fungus *Gigaspora margarita* with *Lotus japonicus* (Deguchi et al. 2007).

Among genes specifically downregulated by EM symbiosis with *P. sylvestris*, the enrichment of GO terms related to monooxygenase activity, metal-ion binding and tetrapyrrole/heme-binding results from numerous putative CYPs in this gene selection (Supporting Table S6). CYPs are widespread heme-thiolate monooxygenases which show great diversity between fungal species and catalytic versatility (Črešnar and Petrič 2011; Qhanya et al. 2015). Their function ranges from the degradation of xenobiotic compounds (Lah et al. 2011), wood-degradation (Ichinose 2013) and plant pathogenicity (Qhanya et al. 2015). Interestingly, multiple transcriptomic analysis performed on fungal AM species at different stages of AM symbiosis identified augmented expression of diverse CYPs genes, indicating CYP proteins may be necessary for AM formation and establishment (Handa et al. 2015; Shu et al. 2016). Identification of a large number of repressed *P. involutus* CYPs genes during EM symbiosis with *P. sylvestris* (Fig. 7f; Supporting Table S6) indicate that CYP proteins may have a negative effect on the establishment and maintenance of EM symbiosis. The different transcriptional regulation of CYPs in AM and EM fungi could originate from the physiological differences between AM and EM formation. For instance, during AM symbiosis, CYPs may be required for penetration of fungal hyphae inside the host parenchyma and/or suppression of plant defence compounds. Alternatively, the presence of CYPs in the Hartig net of EM fungi may result in the production of fungal toxins harmful to the plant host or CYPs may degrade important signalling molecules exchanged between the fungus and its host.

## Conclusions

EM fungi form a symbiotic partnership with host trees, providing an extensive extraradical mycelium that enables trees to efficiently forage and acquire limiting nutrients such as P. Our study firstly explored the transcriptional changes induced by Pi availability in the hyphae of EM fungus *P. involutus*, while growing either in symbiosis with *P. sylvestris* or alone. We provide evidence indicating that Pi starvation and EM symbiosis have an independent effect on the transcriptome of *P. involutus*, with only a small proportion of genes of unknown function co-regulated by both.

Analysis of genes regulated specifically by low Pi availability confirmed the upregulation of putative high-affinity Pi transporter genes as one of the main fungal responses to limiting Pi conditions, whereas the expression of genes encoding organic acid transporters were found to be downregulated. Additionally, the analysis revealed a close transcriptional interplay between the metabolism of essential elements P and N in the fungal hyphae at low Pi availability.

Our analysis revealed both similarities and differences with the transcriptional changes occurring in better-characterized AM root tips, particularly related to the maintenance of mycorrhizal symbiosis. Notably, GTP-related signalling was found to play a positive role in the maintenance of EM symbiosis, as previously reported for one AM fungal species. On the other hand, CYP genes were found to be specifically downregulated by EM symbiosis, whereas these genes were found to be over-expressed in AM root tips during AM symbiosis in different fungal species.

## Supplementary Information

ESM 1 (DOCX 638 kb)

ESM 2 (XLSX 906 kb)

ESM 3 (XLSX 829 kb)
