# Supplementary material for: Phosphate availability and ectomycorrhizal symbiosis with Pinus sylvestris have independent effects on the Paxillus involutus transcriptome
Source: Mycorrhiza. 2020 Nov 16;31(1):69–83. doi: 10.1007/s00572-020-01001-6 (PMC7782400; doi:10.1007/s00572-020-01001-6)
Supplement: Supplementary file 1 — (DOCX 638 kb) [file 572_2020_1001_MOESM1_ESM.docx]

**Supporting Information**

Article title: **Phosphate availability and ectomycorrhizal symbiosis with *Pinus sylvestris* have independent effects on the *Paxillus involutus* transcriptome**

Authors: Christina Paparokidou, Jonathan R. Leake*^,^* David J. Beerling and Stephen A. Rolfe

The following Supporting Information is available for this article:

**Fig. S1** Mycorrhization rates of *Paxillus involutus* on *P. sylvestris* roots at increasing Pi concentrations.

**Fig. S2** Comparison of *Paxillus involutus* Pi transporter expression as assessed by qRT-PCR and RNA-seq.

**Fig. S3** Phylogenetic comparison of putative *P. involutus* Cytochrome P450 genes (CYPs) significantly downregulated by EM symbiosis at any Pi concentration.

**Table S1** Composition of MMN-NS and MMN-S media.

**Table S2** Composition of the FS and 10% strength MMN fungal culture media.

**Table S3** Characteristics of the putative *PiPT* genes and their corresponding proteins.

**Table S4** Gene specific primers used in the qRT-PCR analyses.

**Table S5** List of 3167 unique *P. involutus* genes showing statistically significant differential expression in one or more experimental conditions.

**Table S6** *P. involutus* genes up or down regulated by Pi and/or symbiosis.

**Notes S1** Genomic sequences of the *P. involutus ATCC 200175* *PT* genes.

**Notes S2** Amino acid sequences of the *P. involutus* *ATCC 200175* putative PTs.

**Fig. S1** Mycorrhization rates of *Paxillus involutus* on *P. sylvestris* roots at increasing Pi concentrations. The dot plot shows average EM root tips per 10mg of dry root tissue (n = 6). Error bars indicate standard deviation from the mean. Increasing Pi supply had no significant effect on mycorrhization rates (1-way ANOVA, p > 0.05). Letters indicate output of multiple comparisons between each Pi concentration (Tukey’s post-hoc test, α = 0.05).


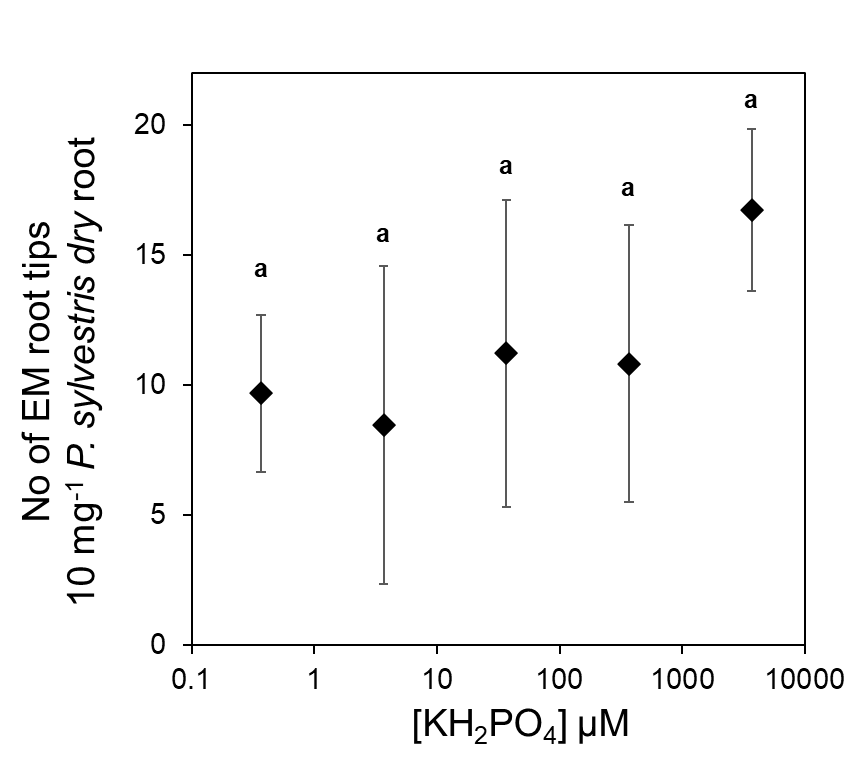


**Fig. S2** Comparison of *Paxillus involutus* Pi transporter expression as assessed by qRT-PCR and RNA-seq. *PT* gene expression relative to actin is shown for individual replicates at two Pi concentrations, in non-symbiotic (NS) and symbiotic (S) conditions. The boxplots show the median value and interquartile ranges with bars extending to 1.5 times the interquartile range.


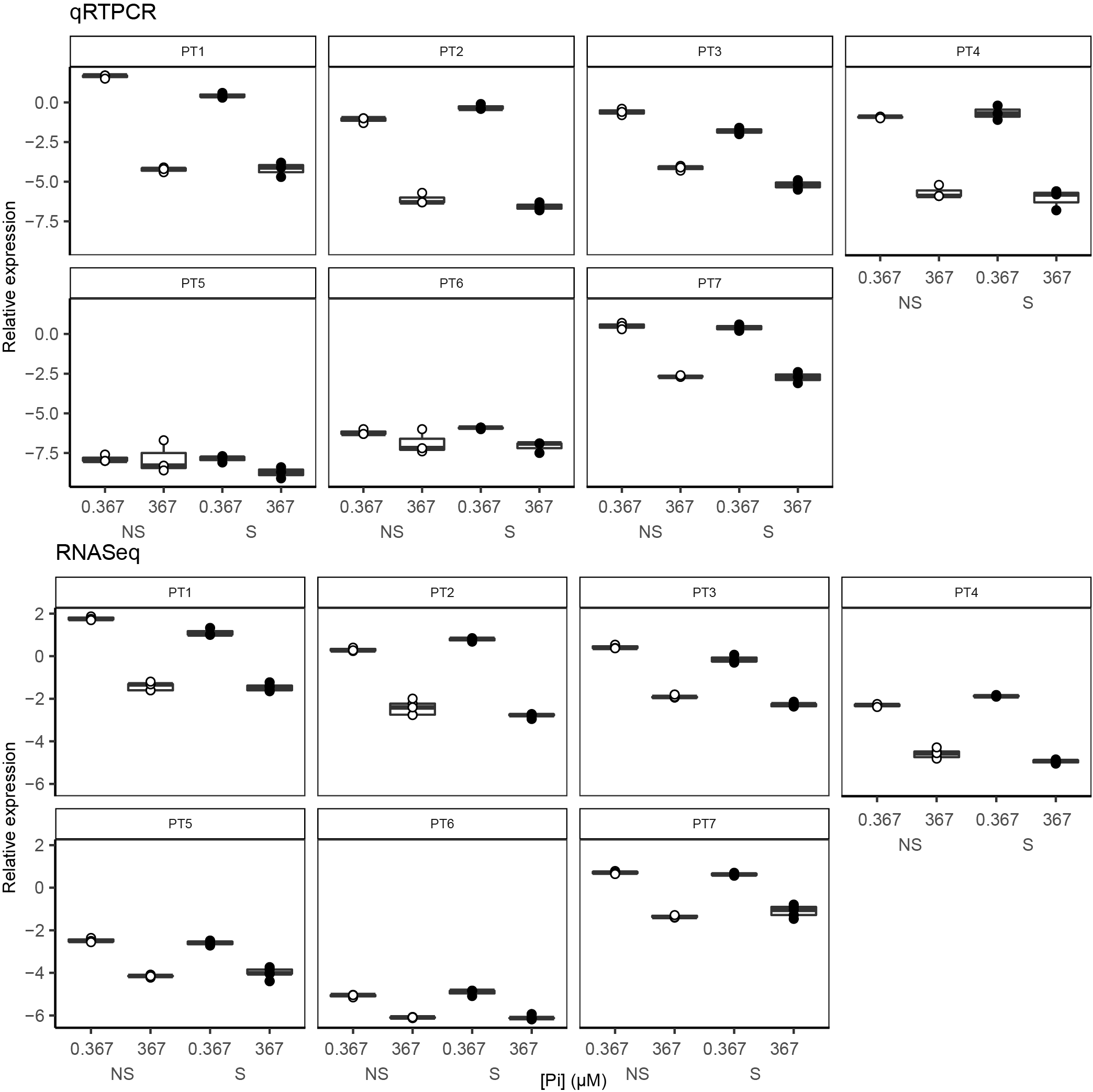


**Fig. S3** Phylogenetic comparison of putative *P. involutus* Cytochrome P450 genes (CYPs) significantly downregulated by EM symbiosis at any Pi concentration. A Maximum Likelihood tree was calculated using amino acid sequences of eight putative *P. involutus* CYPs (yellow) compared with CYPs from different fungal species, functionally classified into primary metabolism (red), secondary metabolism (green), and xenobiotic compound degradation (purple) as described in (Moktali *et al.*, 2012). Gene *gm1.7411_g* (TableS6; EM down) was omitted from the analysis as the amino acid sequence was short and did not overlap with other short CYPs.

**
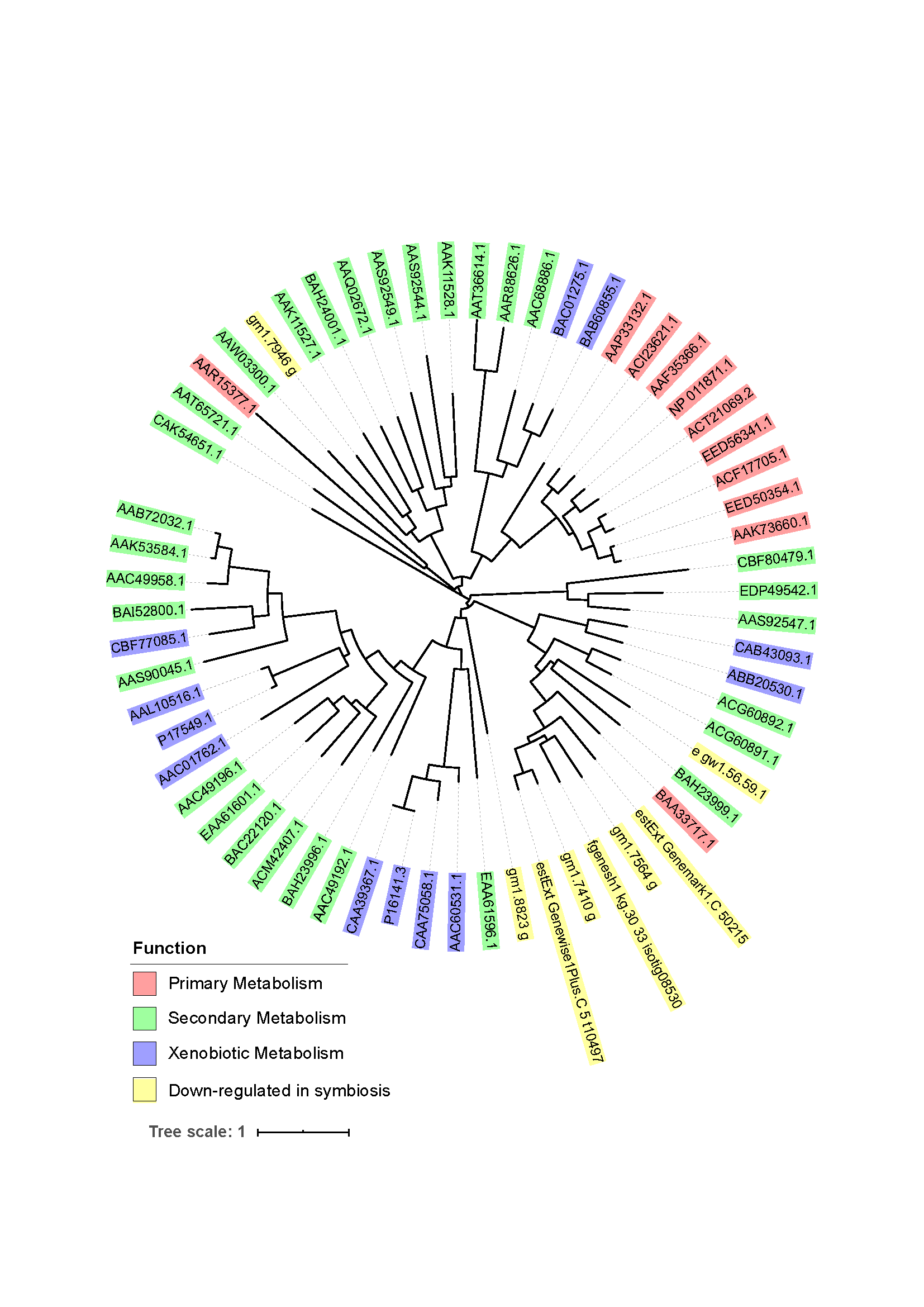
**

**Table S1** Composition of MMN-NS and MMN-S media. Final concentrations in the medium are given. The total volume of the media was brought to 1 L with UHP water. The pH of the media was adjusted to 4.7 for the MMN-NS and to 5.5-5.7 for the MMN-S with 10% HCl. The media were autoclaved at 15 psi for 15 min at 120°C. Stock solutions of 37 mM FeCl_3_.6H_2_O and 3 mM B1 (thiamine HCl), which were filtered sterilized through a 0.22 μm filter (Millex) and added to the media when the medium temperature was ∼50°C.

| Compound | MMN-NS | MMN-S | Supplier |
| --- | --- | --- | --- |
| CaCl_2_*2H_2_O | 0.34 mM | 0.34 mM | VWR |
| NaCl | 0.43 mM | 0.43 mM | VWR |
| MgSO_4_*7H_2_O | 0.61 mM | 0.61 mM | VWR |
| (NH_4_)_2_SO_4_ | 1.89 mM | 1.89 mM | VWR |
| KH_2_PO_4_ | 0.15 μM  0.37 μM  3.67 μM  36.7 μM  367 μM  3670 μM | 0.15 μM  0.37 μM  3.67 μM  36.7 μM  367 μM  3670 μM | VWR |
| FeCl_3_*6H_2_O | 0.037 mM | 0.037 mM | Sigma |
| B1 (thiamine HCl) | 0.3 μM | 0.3 μM | Sigma |
| MnSO_4_*4H_2_O |  | 9 μM | VWR |
| H_3_BO_3_ |  | 46 μM | VWR |
| (NH_4_)_6_Mo_7_O_24_*4H_2_O |  | 146 μM | VWR |
| ZnSO_4_*7H_2_O |  | 153 μM | VWR |
| CuSO_4_*5H_2_O |  | 157 μM | VWR |
| Glucose D+ | 55.5 mM | 11.1 mM | VWR |
| Maltose D+ | 8.32 mM | 2.77 mM | Sigma |
| Activated charcoal | 0.3% w/v | 0.3% w/v | Sigma |
| Agar (Noble) | 1.5% w/v | 1.5% w/v | Difco |

**Table S2** Composition of the FS and 10% strength MMN fungal culture media. Final concentrations in the medium are given. The total volume of the media was brought to 1 L with ultra-high purity (UHP) H_2_O. The pH of the media was adjusted to 4.7 with 10% HCl. The media were autoclaved at 15 psi for 15 min at 120°C.

| Compound | FS MMN | 10% MMN | Supplier |
| --- | --- | --- | --- |
| CaCl_2_.2H_2_O | 0.34 mM | 0.34 mM | VWR |
| NaCl | 0.43 mM | 0.43 mM | VWR |
| MgSO_4_.7H_2_O | 0.61 mM | 0.61 mM | VWR |
| (NH_4_)_2_HPO_4_ | 1.89 mM | 0.189 mM | VWR |
| KH_2_PO_4_ | 3.67 mM | 0.367 mM | VWR |
| FeCl_3_*6H_2_O | 0.022 mM | 0.022 mM | Sigma |
| Glucose D+ | 13.87 mM | 5.55 mM | VWR |
| Malt extract | 1% w/v | 1% w/v | LAB M |
| Agar (Noble) | 1.5% w/v | 1.5% w/v | Difco |

**Table S3** Characteristics of the putative *PiPT* genes and their corresponding proteins.

| Gene model name | Protein id | KOG description | Gene name in this study | Number of exons | Theoretical (Av.)  pI /Mw (Da) | Predicted subcellular localisation |
| --- | --- | --- | --- | --- | --- | --- |
| fgenesh1_kg.22_#_22_#_isotig08265 | 169850 | Inorganic phosphate transporter | *PiPT1* | 10 | 8.19 / 57,920.28 | Plasma membrane |
| gm1.13401_g | 18830 | Inorganic phosphate transporter | *PiPT2* | 16 | 8.54 / 52,279.79 | Plasma membrane |
| fgenesh1_kg.1_#_253_#_isotig09075 | 165085 | Inorganic phosphate transporter | *PiPT3* | 17 | 8.35 / 53,781.42 | Plasma membrane |
| estExt_Genewise1.C_14990003 | 141142 | Inorganic phosphate transporter | *PiPT4* | 7 | 7.60 / 22,700.48 | Plasma membrane |
| fgenesh1_pm.14_#_104 | 163069 | Inorganic phosphate transporter | *PiPT5* | 17 | 7.69 / 40,312.56 | Plasma membrane |
| fgenesh1_pg.404_#_2 | 158250 | Inorganic phosphate transporter | *PiPT6* | 10 | 6.25 / 39,169.09 | Plasma membrane |
| gm1.7522_g | 12951 | Inorganic phosphate transporter | *PiPT7* | 13 | 6.30 / 28,898.48 | Plasma membrane |

**Table S4** Gene specific primers used in the qRT-PCR analyses.

| Gene model name | Protein id | Gene name in this study | Primers |
| --- | --- | --- | --- |
| fgenesh1_kg.22_#_22_#_isotig08265 | 169850 | *PiPT1* | Fwd: 5’- CAGCTCGGTTTCGGTCAA -3’  Rev: 5’- GATGAGCAGAGTCGAGAAGATAC -3’ |
| gm1.13401_g | 18830 | *PiPT2* | Fwd: 5’- TTTCGGGTGTAGCATCGC -3’  Rev: 5’- AGTTCTTCCGCAAGGATCG -3’ |
| fgenesh1_kg.1_#_253_#_isotig09075 | 165085 | *PiPT3* | Fwd: 5’- TTCTGCAGCCATCGGTAAA -3’  Rev: 5’- CAAATAGCGGCGATGATGAAAG -3’ |
| estExt_Genewise1.C_14990003 | 141142 | *PiPT4* | Fwd: 5’- CCTTGCGACTATCCTCTGTATC -3’  Rev: 5’- CCGAGGAGAATGCGAAAGATAG -3’ |
| fgenesh1_pm.14_#_104 | 163069 | *PiPT5* | Fwd: 5’- CCTCTCTCCTATGACGGAATGA -3’  Rev: 5’- TTCTCTTCCTCGGAGATCGT -3’ |
| fgenesh1_pg.404_#_2 | 158250 | *PiPT6* | Fwd: 5’- TTTCGGGTGCTGCATCG -3’  Rev: 5’- TTCTGCAAGGATCGCATCG -3’ |
| gm1.7522_g | 12951 | *PiPT7* | Fwd: 5’- ATTCTTCCTTGTTAGGCCTCTG -3’  Rev: 5’- ATGTGTCAAACCCGTGCT -3’ |
| gm1.14674_g | 20103 | *ACTIN* | Fwd: 5’- AGTCCGGTCCTGGTATTGT -3’  Rev: 5’- GACTTCTGTATCGTCCCTCTCT -3’ |
| e_gw1.37.38.1 | 82237 | *UBIQUITIN* | Fwd: 5’- CGCACCCTTTCTGACTACAA -3’  Rev: 5’- AACATCCACCACGCAGAC -3’ |

**Table S5** List of 3,167 unique *P. involutus* genes showing statistically significant differential expression in one or more experimental conditions.

TableS5.xlsx

**Table S6** *P. involutus* genes up or down regulated by Pi and/or symbiosis.

TableS6.xlsx

**Notes S1** Genomic sequences of the *P. involutus ATCC 200175* *PT* genes.

>*PiPT1_* fgenesh1_kg.22_#_22_#_isotig08265

TGGCAGAGGCCAAGTAGTTGCGACGACGCGCAAGGTCCCTGGCAGATGTGCACGTTCTCAACGAAAGTAACTGATTTCGGAACACGGGAGGCGGGTCAACAAATGAAAGGAAGAGCATTCGTCGGTAAGTTTTTTGGGGTCGGACGACGTTTATCACGGTGACTGATGGGCCGGGTACGATTACCGAGGGAAAGCAGGGGCAAATCTTTGACCTGATCAGACTGCACATTGCAGAAAGAAGACGCCTTAAACGTGCAACGAGAATCTTGTATTAGTCAGCATCATTCTCACTCCGATTGGCCAATGGCCACAAACGTGCAAGGGGATGGTTCCTTAGCCAAGCCGAGTGGTTCATTGATCAGAGTTTGATCAGGGGCACGCTCATTTCGGTATCCGGTTATAAAGCTTCGATGAACTTTGTACATCCTAAGCGCTCTGTCCTCCCCTCACTTCCCTTGCCTATCCTCGAGGTTTCTAGGGATCAATGGCTTCCAACGCTGACAGACTCTCTGACCGCGCCTCATATGAGAAGAACCTCACACCTGCTAACCTCAATGAGCGTCGTCGCGCGGCTTTGCAAGAGATCGACAATGCTCCATTCTCGTATGTTCCATTCGACCACCAACCGTTCATCTCACCTATTTCTTCTCCCCCCCTCTCAGGTGGTTCCACGTAAAGGTCTGTTTGGTCGCAGGTGTCGGCTTCTTCACCGATGCGTGAGTCCATAAAATTTATTTCAATCAAACGTTCCTCGTAACATGGTCATCCTTCTAGCTACGATATCTTCGCTATCAACATCGCTGTCACCATGTTGGGCTACGTATACGGTAAAAACGGTATGTCGCCAGACAATATTTTTAATCTCCTCCGTCCTTTTCATCAAACAATCTTTCCGTTTCTAGCCGCCCTTACCCCCTGGCAGTCGACCGGTCTCAAGGCTGCCACTCCCGTTGGAAACTTGGTTGGTCAGCTGGTGTTCGGATGGCTGGCCGATGTCCTTGGTCGTAAGCGCATGTGTGAGTAATCGAATTCCCCCATAACAAATGTCCACCGCTCACCGTAACAATCTTTAGATGGTATCGAGTTGATGATCATGATCATCGCCACCTTCGCACAGGCTGTATCTGGTGAGGCCCCTGCTATCAGCATTGTCGGCGTCCTCATTGTATGGCGTTTCCTGGTGAGTCCCCCCTTACCTCAAATCCCCCAGGAAATCTGATCATGGCCTCATTAGATGGGTATCGGTATTGGAGGTGACTACCCTCTTAGTGCCATCATCACGTCCGAGTTTGCAGCTACACGCTCCCGTGGTCGTCTCATGACCGCTGTCTTCGCTGCACAGGGTTGGGGTCAATTAGGTATGCTCAGCATCCCTTTCAGGTCTCTAGTCTGATTAACCGCGAATTAGCTGCTGCCCTTGTTGCTCTCGTCGTTGTTTCTGCATACAAGGAGGCCATCCTCGCTGGTCCATTCCCTTCCGCTCTCCCTATTGATCATGCCTGGCGTCTGCTCATCGGTCTTGGATGCGTCCCCGGAGTTGTCGCTCTTTACTTCCGTCTTACCATCCCCGAGACTCCTCGTTTCACAATGGATGTCGAGCGAAATGTTGCTCAGGCTTCGCAGGATATTGAGAACGTACTCACAATTGGCAAACACGTCGAGGCCGAGGACGCTGTTGTGGAGCGTGCCAAGGCACCCAGGGCAACCCGTGCCGACTTCGTCGCTTACTTCTCCAAATGGGAGAACTCGAAGGTTCTCGTCGGAACGGCCTACTCCTGGTTTGCCCTCGACGTCAGTGTGATCCGTTGCTTTGTTCTACTCTGTGTCATCTGAGATGTTTCCAGATCGCGTTCTACGGCCTTGGACTTAACAGCTCCATCATCTTGAGTGCCATCAAATTCGGTGGATCTACCGCAAAGAAAGGCACTACTCTCAATGTCTACGAGAGTCTCCACAATATCTGTATCGGAAACATCATCCTTTCCGTTGCTGGGCTTATCCCCGGTTACTGGGTTTCCTTCCTTTTCATTGATACCTGGGGTCGCAAGCCCATCCAGCTCATGGGCTTCACTGCCCTGACCATCTTGTTTGTCATCATGGGTATATCTATCACCTGTCGTTTTCAGACAACACAGCTGACTCCTCCCCTCAGGCTTCGCATATGACAAGCTGGTCGCAACGACCTCTGGCACCAATGTTTTCGTCTTCCTCTATTGTCTCACCAACTTTTTCCAGAACTTCGGTCCTAACACTACCACATTCATTGTCCCCGGAGAGGCTTTCCCCACGCGTTACCGTTCGACCGCGCACGGTATCTCCGCTGCAGCTGGCAAACTCGGTGCCGTCGTTGCGCAGCTCGGTTTCGGTCAACTGGTTAACATCGGAGGCACGAATAAATTTGTGAAACACATGTGAGCATCATATCTGTTCCCTACCTTGAATACCATTCTGAAAGATCGCTCTCGCACAGCATGGAAATTTTCGCGTTTTTCATGTTGACCGGTATCTTCTCGACTCTGCTCATCCCCGAGACCAAGCAGAAGAGCCTTGAAGATCTCTCAAACGAAGACCAGGCGGAATACATAACCGATTCTTAAGGCAATGGAAAAATAGCCAGTATTTATGTAATACGCTGTGCACTTTTCGTTTTCTCATCTTGGAGGGCTACATTTTGCTGTTGTAATTACATTTTGTGCGCCACTATTAAGGAGGCGCACATCGGACTATAATATTCCGAATAGTCACGGTGAACCTAGTGTGTTAGCGATGACGTAGTTATTTAAGTCATCCTCTTGTGTCACGGTGTACCTTTGCCAAGACTGTTGTATCCCGACGGTGACTAGTGTCGTTCTTAGCGTCGAAGAAAAGATATTCATCATGACACCCAAAGGCAAGGATCTTCCGCCCCCAAGTTTCCCAGCGCCCACTGAACCTAGCGAAACAGTCCCAAACCCAGGCGACAAGCAGGCCAGAAAGTC

>*PiPT2_* gm1.13401_g

GACTCTTCTGATGACTTTCCTGACGTCTTTCTCCGCACCCAGGTCGGATTCTTCCTAGATGGTTCGTTCATCATACTTTCATCAAACTAACAGACACGTTAACGAAGCCCAACCAGCCTATGACTTGTTCATCATCAACGTAAGCGGGGTTCTTAGCGTCCGCTCCATCGCCATCCATTTGACCGTTTCCATAGCCCGTTGCGACCATGTTGCAATACAGGCTTTACGGAGGACAAAGTCTTCCCAACAACCTTCAAGGTGTCCTGAAAGCTTCTGCGAACATTGGCAGTGTCATTGGTCAATTCCTTTTCGGTAAGTAAATATACAGAGGTGTATCACTCCACAGCCTTACCAGTTCTGCTCAGGCTACTGCGCCGACGCTTTCGGCCGGAAGGCCATTTGTATGTACCTTCGTTCCGTCTCCTAAATTGATGAAGGGCTGACGTTTATCCGAAGACGGAAAAGAGCTGATGCTGATTATCCTTGCGACTATCCTCTGTATCTCATGCCCAACAAGTAAGCACGGGCATCAACCCTATAGCCACGGAAGCACTGAATGAAGTTATTTCATAGATCTTCTCTCCCCCGACGGATCTTTGATATACTTATCTATCTTTCGCATTCTCCTCGGAGTCGGTGTTGGAGGGGATTACCCCATGTCAGCTTCCATCACCAGTGATCGCGCCGTCCTCCGCAAGCGAGGGACCCTTCTAGCGTACATTTTTGCTAATCAAGGATGGGGCAGTTTAGTCGGAAGCTTGGCGACAATCATCGTTCTGGCGTGTTACAAACATGTTATGAACGATGAGGGTGAGACCTCGAAAGTCGATGGGGGTAGGTGGCGCTTTAGCATTTTTGCCTCACGTTGTCTAAGCTCATACTTGTCCCATAGTGTGGCGAATCGTCGTTGGTCTCTCGCTCATCCCCGCCTTCGGAACTTTATACCAGCGGTTAACGTTGCCCGAATCTGTACGCTTCGAAGAATCGCGAAAGCTGACCGCGGGAGAACTCGACGATTTAAAAGCAAAGAACGATGTCGAAATCACTGTTTCAAGCAAGAACTCCAATGAGGGTCACGTCAAAGAACCCTTGGAGAAGAAAGCCCACTTCAAAGGTGACTTCTTCCCGCATATCACAGGGGTAGTTGCTCACTCGCTTCGTCCACAGAGTTTGTCGAGTACTTCTCCGAGTGGCGTCACGCGAAAATATTGATTGGGACGTGCACGTGTTGGTTCTTGTTGGACGTTGCGTAAGTTCTTGCTCCTGTCACGTCTAGCAGTGTCTGATTTGTTGGTTTGTCCCAGGTTCTATGGCATCAACTTGAATCAAAACGTGGTACTTCAACAAATTGGGTACGCTGGTAAGACAGGTACAGCATGGGAAAAACTTTTCAAAGTGTCTACTGGAAACATTATCATCACCGCTCTTGGTTTCGTTCCTGGTACGTACGGCTTGTAGCTGACCCACCACATCAACCTTCTAACTCCCGCTCTACTACTACAGGTTACTATGTGACCGTCCTCACCATCGAGAAGTTGGGCCGCAAGTACATTCAGATCCAGGGATTCCTTATGACTTCCCTCTTCTGTAAGTTTCGACTCGCTCGACCCCATCCCAAATCCTAAATCTCAAATCATCTTCTTCGAAAGTGGGCATATTGGCAGGGAAGTTTCACACCTTCGACAATGCGTCGTTCATAGTCTGCTTTTCGTTCCTGCAGGTGAGCCCGCCTTTCATTTCTTCGCGTTCAGCCACTCTAACACGAGACCAGTTCTTCTTCAACTTTGGAGCCAATGCGTAAGTAGCTGAGCACACCGTCTTGCAGTTCGTCCAACTGACTGTCTCTGCCCCCACCGATAGAACCACATATGTAAGCAACGCTCTCATGGATGACTGTAACCTTGTCTTAATCGCACGCTCAGTGTTACCCCGCCGAAGTATTCCCTACAAAATACCGTGCATCTGCACACGGTCTCTCTGCTGCCTGCGGGAAAGCAGGCGCCATCATATCTGCTCTCGGTTTCAACACGTTAACCGCCAAGATCGGAACGCCAGCCGTTCTATGGAGTCAGTACACTTCTTGCTAACCGCCTTTGACACCGTATCTAACCCACACACTCGTGTTATAGTCTTTTTCGGGTGTAGCATCGCAGGAGCAGGTTCGTCACGTCCATTGGACACGTGTTGAACCATCATTCATGTGATTTCCGTTCACAGGATTCACGCTCCTCCTTCCGGAAGTCGCTGGACGCGATCCCGACGCGATCCTTGCGGAAGAACTCAGGGAACGATAAACGACGGATCGTCCCTCGAGCTCCGTTCTAAATCGAGAACGAAGAAGTCACCAGTCACCCATGTCCTCTTTTCTTACTATCTCAGAAATCATATTTGACTCATCCCTAGTATCTATCCGTTATTCGAAATCATGTGGTCTAATCGTGGCCTGCTGTTGTTGTGGGAATGGGTGGTGGTGGGAAGTCCGAGTCGTCGCTAG

>*PiPT3_* fgenesh1_kg.1_#_253_#_isotig09075

ATGCCGCCGAACATCAATGTGATCCATGGCGGATCTTAACAGCGCTAATCGCTGTCTATTAGTTCCCTACATACTCCCGATAGCACGTGCGTCCCCTCTATAAAACGCATTCCCAGTAGCATGGATGGACGTCCTTGTCTTACGAGAACCCCCCTCCCTTCTGTTCTCGGCCTTCCCTGGATAACTCTCAAAGTTTAATTTTCTGGTACACTTTTCTCTCATGTCGGCCGAGAAAGACGTCCCCACCCTGGAGGCGGAACAGACCACTGATCCCCGAAATGTCTCTGTCAAGCCTCGCGAGACTAATCTCATTATTGCACCTAACAATCTTGCCAAACGAGAATCTCTTAGCGCTTGGCTTACTATCTGTGCAGCGGGCTTTGGCTTAATCAGTGATGGCTGTAAGTCACAGAGTTTCGTGTCAGCGCCGGATTCAAGTTAATATCCGTCCTTTAGATCAAAACAACCTGATGACCATGTCAAATGTAGGCCCATCCCATCCCCCCCTGCTTGTACTCGTCCTAGAGTAGTTCTCTAGGTCATCTTCAAGACACTTTACCCCAAGGACTATACTTCCGCGGTCTCTACGCGGGTATCCAATGCTCTTCTTGTCGGTCAGTTCTTATATACACAGATGCTTCTTTGTAAGTTTGTCTAAACTACACCTCTACCAGGCGCCGTCATCGGGCAAATCTTCGTTGGCTTGATTTGCGACAGAGCCGGTCGCAAGGTTGCCCTTGTTGCCACTACCTTGTTCATCGTTCTCGGTAGTACTCTCGGAACTGCCGCCCATGGTGCTAACGGAAGTGCACAAGGCCTCTTTTGGTTTTTGACTTTCGCCCGTGGAATCACTGGCATCGTAGGTCATTCATCCATTGCACGTGTTTCAGTTGCTCAAATAATTTTTGCAGGGTGTCGGTGGAGAATACCCCGCTTCGTCAACAAGCGCTAGTGAAGCCGCAAACGAGAAAACACAGAAGAATCGCGGTCCAGGTATCCTTACCATTGTTATTTTCCGCCTCAACCATTCACTGCTAACATTCCTCCCAGTCTTTATCATGGTCACAAATTTCGTGCTCTCTGTACGACCTTCATTCTTTCGGGGTCTCTGCGAACTAATGAAACACCTCTGAATTAGTTTGGTGGCCCATTGGCTGTTTCAGTGTTCTTGATCGCACTCTCAGCAGCCGGGGAAAATCACCTTCAGACTGTCTGGCGCGTTTGCTTCGGCGTCGGTATACTCTTGCCTTTGACAGTGCTCGTCTTTCGTTTACGGATGTTGAGCTCCAAACTCTACCGGAAGGGTGCCATTAAACGTAATCTGACATTCAAACTTCGCCTTGGAAGTAGCACTCATCTGTGAGCCAGGTGACGTTCCCTACGGGTTGGTGATAAAACGCTACTGGCGTCCTCTCATCGGTACCTGTGGAGCGTGGTGCGAACACATGTTTGTACAACTAAATGATGGCTAATATGTTGGTCAGGTTCCTTTATGACTTTGTATGCCTTCACTCCCGAGTTCCTGAACTTTTCAATGGCTTGACTGATCTCTTAGGTCACTTTTCCGAACGGCGTGTTCTCGGGAACTATCATCTCCAGTGTGATCCACAACGGCGATATCAAGAAAACTGCTGAATGGCAGCTTTTACTCGGAGCAATAGCCCTGCCTGGCGTCCTCGTCGGCGCTTGGCTCTGCAACCGACTGGGGAGAAGGAACACAGTATGCGACACACGACCCGCATTCATTCAAGCTACTCATCCTCTACCCCTTAGATGATTTTAGGATTCTCTGGATACCTAGTATTTGTAGGTCTTTCTCCTGCTTCCAGCTCATCATTTGCTACAATTGGCACGCCAGGGCCTCATAATTGGTCTCGCATACGATGAAATAACCAACATTATACCCCTCTTTGTAATTTTGTGGGTGTCATCAAAAATCCTTCACTAAACTGAGCTGAGAGCTGTCTTGTAGCTATGGGCTGATGCAGTCGTTTGGTAACCTGGTGCGTCACACTCGAGGTCGATCAGCTTAGTTGTTCAGATGATTACAGGGACCTGGTGATATGCTGGGCCTGACGAGTTCTGAGTAAGTCCATCGCAACTCCCTATTTACCTTGAAACTTAGGGGCCACACCACAGGTCGTATGCAACGCCCGTTCGGTATGACGCTAGCTCACGTTGAGTTGAAGAGACTCAATTTGATTGTCAGTGGAACATGTTACGGCCTTTCTGCAGCCATCGGTAAAACTGGCGCTGCTATTGGGACCCAAGCATTCACGCCTATCCAGAATAATCTCGGAAAACGGTACGAGTACTTCCCTTGCTTTCTTCCTTCTTGCTGATCCAGACAATGAAGTTGGACTTTCATCATCGCCGCTATTTGCGGTATTGCTGGAATCCTTGTGACGTACTTCTTCGTGCCTGACATGACTGGCATTGACCTCGCGGACGAAGATGCCAAATTCATGAAGTACCTCGCAGAGAATGGTTGGCAGGGCGAGGTTGGAGAGGACGACGACAAGGCGTTGATTGGCACATATGCCAGTGAAGACGCCCTGGAGGAGAAGATCTGATATGAGATTAGTATATGTCCGTCACGATGGTATCATATGGGTAGTTCACCGTCTTTGCAACTTTTGAATTGGCGGAGGGTTGAAGATCACGTGCGCACTGAGGATTTCGCGTATTACTACTATTAGACTACAGTTGCTCAGCACATCTTGCCTTGAATGTCTGCTCAGGTTCTCCGGCAGTGCGCGTCTCGTCGTTCTTTGTGTTTCTCGTGTCTACAGTTTCGCCGCGCCAAAGCATACTACTCGCAATCAGCTCTCGCTTTTTCCTCCACCA

>*PiPT4_* estExt_Genewise1.C_14990003

AATTGATGGGCGTGCGATGCGCCCGCTCCTGCCGTGCGAGTCGACAAACGTGCAATGAGCGTTTGATGAACCTGGCGAAGGGCAATCAAGGTTCATGAACGTTCGACCTGACTTTGGACAGAGCCATGGCACATGTCAATTGCACGCCACACGTGCAACCGGGCCAAACCCATCTTTATCCTACTCCCCATGCTCGTGTGATCACTCTTTCCAGCTTCCCTGACTCTGCCCTCAGTCACCGCAATTTGGTCTACCCTTAGATTGTCATCGGGACAGTGGAACACGCGGAGATTCCCCGTTTCGAGTTTGGGCCGTTCTCCGTTAACGACCTTGCCGAAGAAGTCGGCCGTCTATTTGCTCCATGGTGCCCATAGTGCAATCTTATAAATTGAAGAGCTGACACCATATCTTTACCTGTCTCGTCCTTCTTCTCCAAGTCTGCCATGGCTAGTCACATCGAGGAAAAACCAATCTCTTCTGCTGAGACTGGGACCCCGTCGGCCCCTTTTCGCTCAGAGCTTCAGGCGTATGAGCGCGATGCTGGGGATAGGCCCCCATACGTTTTGACCTATCCTGAAATCAAGCTCTTGGGTATTGCCGGGGTGAGTACACCTAGTGACTCTTCTGAATGACTTTCCTGACGTCTTTCTCCGCACCCAGGTCGGATTCTTCCTAGATGGTTCGTTCATCATACTTTCATCAAACTAACAGACACGTTAACGAAGCCCAACCAGCCTATGACTTGTTCATCATCAACGTAAGCGGGGTTCTTAGCGTCCGCTCCATCGCCATCCATTTGACCGTTTCCATAGCCCGTTGCGACCATGTTGCAATACAGGCTTTACGGAGGACAAAGTCTTCCCAACAACCTTCAAGGTGTCCTGAAAGCTTCTGCGAACATTGGCAGTGTCATTGGTCAATTCCTTTTCGGTAAGTAAATATACAGAGGTGTATCACTCCACAGCCTTACCAGTTCTGCTCAGGCTACTGCGCCGACGCTTTCGGCCGGAAGGCCATTTGTATGTACCTTCGTTCCGTCTCCTAAATTGATGAAGGGCTGACGTTTATCCGAAGACGGAAAAGAGCTGATGCTGATTATCCTTGCGACTATCCTCTGTATCTCATGCCCAACAAGTAAGCACGGGCATCAACCCTATAGCCACGGAAGCACTGAATGAAGTTATTTCATAGATCTTCTCTCCCCCGACGGATCTTTGATATACTTATCTATCTTTCGCATTCTCCTCGGAGTCGGTGTTGGAGGGGATTACCCCATGTCAGCTTCCATCACCAGTGATCGCGCCGTCCTCCGCAAGCGAGGGACCCTTCTAGCGTACATTTTTGCTAATCAAGGATGGGGCAGTTTAGTCGGAAGCTTGGCGACAATCATCGTTCTGGCGTGTTACAAACAT

>*PiPT5_* fgenesh1_pm.14_#_104

AAAGGTCAGGTGAGTTAGCGAATCTGCTGAGTGCTCATCCCCTGAAATTGTAAATGAGATTTACCACACCCTGGTTACGCCCTTAAAGATTCTTGAAGCAAAGCATGACCCCCGGAAACGGTTATAAGAAGCAGCCATGTTGGACGCGACCACTCGCTCCTCGCTCCTCTGTTCAGTGTTGACTTTGCTATCCACCAGAGATGCCTGGCTTGAAGGATCTATCCAGCGTTTCCCACATCTTTGCGTAACCTAGTCCTCCTTCTGCAGTGTTGTCAATTCTTGTCATTATTGAACTCCCCGCGCGCTCAAACAGTTGCGGAACAGCCTTATTTTCTGATGGTGCCCAGGTTTCTCCTTGTACTCGTGTAGAGTGATGCGACTTACTTTCTGTGTCCAAACAGGCTACGCTAACAGTGTCATCGGAGCGGGTATGTTTTCAGCCTCCTTATGTTCCTTCCCGTCTCATCTTTCCAACTATAGTCAATACTCGTAAGAATACCCACTGAATCGCCTGCTATCTAGGCTAATCACACCACTTCAGTTTTGGAACGTATCTACGGTTCCGATGTCATGACGGCGAATAACCATAGCAAGGCTTTCACTAGCGTCACCTTCGCAGGTTCAGTCATCTATGCCCTGGTTCTTCCATCATTGCCAACTCACAACCATAGGCACTGTTGTGGGCATGCTTATATTTGGTTGGGTATCCGACAAGATCGGCCGCAAGTTTGGAATGGTGAGCATACCCGTTACAAGTCACTTGGTATCCCTCTTTCCCTTACTCTATGAATTCCCTTAGATATCAGCAACCGGTATTGTTGCTCTCTTCATCCTGTCTGCTGCTTCTGCCGGCGCAAACAACAGTGTGGCTGGAATGTTGGCTATGCTTGCCGCCTGTCGGTAAATATACATTTTGTATGTCAGGCACCATCTCATCATTTTTTTGTTTCTTTTGTTTTTCATCTATAGCTTCTTGATCGGCATCGGCATCGGCGCAGGATACCCCTGTGGAACTGTTTCTGCCTCGGAACAGTCAGAGGAAGGCGCCATCGCCAAGAATGCTCAGCATCGGTGATTGGTACTTGCTACAAGTTCGTAATTCGCAGCTCTACCCCGACTCTCAATAAACATGCTGAACATGATTCTAGATACGATGATGGATGTTGGTTACGTGATTGGGGCATTTGTACCTCTCGTTTTGTGTTGGACGTAAGTCGTATTGCATCCCTGAGACAGGAGCGCTCGAGCCCACTACAGCTCACCAGCTTTGGAAACAATCACATTCGGGCTGTCTGGCGCCTCTCTCTCGGTCTAGGAGTCGTTCCAGCTCTGGCCGTACTTCTTTGGCGGCTACGCATGACGGAGCCACTGAGCTACAAGGACTCCATGAAATCTGCGCGGATCCCATACTGGCTCGTCGTGAAGCGTTATTGGAAAGGCCTCTTTGCACCTTCGCCTTCTTGGTGAGTTGGAAAAGTACCTCGCGGATGGATACCACATTAAACGTGCGCAAAACACAGGTTCACCTATGATTTCATCTCGTACGTGCGCTTGCTTTGTCTTGCAACGGTGGTTCTGAGACCTACATATCTTCCACGATCACCAACAAGTGAGATGTCTCTTACACTCGATAGGTAATTGCATTACTGAGGCCATGGATCTTACAGTGTCACTGGAAACAACACCTCTTCGATAGTCGTGTTCGGCTGGAGTGTTGTTGTCAAGTCACCTCCTAGTCTTCTTGCCTGTATCGTAAAACTCACGCATCTGGCTCCAGTTTGTTCTACATGTCAAGTTCGTTTTTAATCAACCTCATTCTTCCGTATATTCAAGGATATCGCAGGCACCATCATCGGTGCCTTCTTAGTCGACTACTGGGGCCCAAAAGCAACGATGGTAGTCGTTCATATTTCGTAGCAATAATGACAACCCAGACGCTCATTCTAGATCGCTGGTCTACTTGCTCAAGCTGTCACGGGTTCATTGTGAGTGATATATACACTCAACTCACCAGCAACATCGCTGCGTTCGCAGTAAGTTACGACTAGAACGTACAATCGCTATTCTCAATTGAACCTTAATCGCAGATCGTCTATGGGATATTCTTGAGCTTTGGAGAGTTTGGTAGGTCACTCATCGAAATCGTCAAAGCGAATAGTCTGGCCCGACATCATCGCCGGTAATTACTTGTGATAATTGAATCTAACTCGGCCGAGCTCACAGGTTATATGCAGACTTGGGTGGCGATAAGACGGCGAAAGGTAACACGGGTCCCTTCTTGGTCGGAAGTGGACTCGCCATCCTCAGTGCCTTGGTCGTATTCTTCCTTGTTAGGCCTCTCTCCTATGACGGAATGAAGGAAGAAGATGTGAAGGTACTTCCTCGGTCAAGGTTCATCGATGAGATGTATCTGTTCTGACATCTGATGGCGCAGTTCCGTATGCATCTCGAGGGGCCCGGATTTGACACTTCATTAATGGGTGTTCCTGACTCCGAGGTTTCGTCCACGATCTCCGAGGAAGAGAAAGCAGCTCAGACTGCTTGATGAATTGGGCAAAGGGCCCGGCATAGCAAGATCTCACACTTTGGACGATGATTCACGATCAAAAGCAATGTAGGTTGTCTCGGTAGATGCAATTATCTTGTGCTTTCAGCTCTCTGGCACGTACCCGTAGCACTTGGTTAATAAATGGGTATATTGTTCTTGGCTGGCTGGCTCGAGTGAACAATCGACTTTTGATTGCT

>*PiPT6_* fgenesh1_pg.404_#_2

ATGCCGCCGAACATCAATGTGATCCATGGCGGATCTTAACAGCGCTAATCGCTGTCTATTAGTTCCCTACATACTCCCGATAGCACGTGCGTCCCCTCTATAAAACGCATTCCCAGTAGCATGGATGGACGTCCTTGTCTTACGAGAACCCCCCTCCCTTCTGTTCTCGGCCTTCCCTGGATAACTCTCAAAGTTTAATTTTCTGGTACACTTTTCTCTCATGTCGGCCGAGAAAGACGTCCCCACCCTGGAGGCGGAACAGACCACTGATCCCCGAAATGTCTCTGTCAAGCCTCGCGAGACTAATCTCATTATTGCACCTAACAATCTTGCCAAACGAGAATCTCTTAGCGCTTGGCTTACTATCTGTGCAGCGGGCTTTGGCTTAATCAGTGATGGCTGTAAGTCACAGAGTTTCGTGTCAGCGCCGGATTCAAGTTAATATCCGTCCTTTAGATCAAAACAACCTGATGACCATGTCAAATGTAGGCCCATCCCATCCCCCCCTGCTTGTACTCGTCCTAGAGTAGTTCTCTAGGTCATCTTCAAGACACTTTACCCCAAGGACTATACTTCCGCGGTCTCTACGCGGGTATCCAATGCTCTTCTTGTCGGTCAGTTCTTATATACACAGATGCTTCTTTGTAAGTTTGTCTAAACTACACCTCTACCAGGCGCCGTCATCGGGCAAATCTTCGTTGGCTTGATTTGCGACAGAGCCGGTCGCAAGGTTGCCCTTGTTGCCACTACCTTGTTCATCGTTCTCGGTAGTACTCTCGGAACTGCCGCCCATGGTGCTAACGGAAGTGCACAAGGCCTCTTTTGGTTTTTGACTTTCGCCCGTGGAATCACTGGCATCGTAGGTCATTCATCCATTGCACGTGTTTCAGTTGCTCAAATAATTTTTGCAGGGTGTCGGTGGAGAATACCCCGCTTCGTCAACAAGCGCTAGTGAAGCCGCAAACGAGAAAACACAGAAGAATCGCGGTCCAGGTATCCTTACCATTGTTATTTTCCGCCTCAACCATTCACTGCTAACATTCCTCCCAGTCTTTATCATGGTCACAAATTTCGTGCTCTCTGTACGACCTTCATTCTTTCGGGGTCTCTGCGAACTAATGAAACACCTCTGAATTAGTTTGGTGGCCCATTGGCTGTTTCAGTGTTCTTGATCGCACTCTCAGCAGCCGGGGAAAATCACCTTCAGACTGTCTGGCGCGTTTGCTTCGGCGTCGGTATACTCTTGCCTTTGACAGTGCTCGTCTTTCGTTTACGGATGTTGAGCTCCAAACTCTACCGGAAGGGTGCCATTAAACGTAATCTGACATTCAAACTTCGCCTTGGAAGTAGCACTCATCTGTGAGCCAGGTGACGTTCCCTACGGGTTGGTGATAAAACGCTACTGGCGTCCTCTCATCGGTACCTGTGGAGCGTGGTGCGAACACATGTTTGTACAACTAAATGATGGCTAATATGTTGGTCAGGTTCCTTTATGACTTTGTATGCCTTCACTCCCGAGTTCCTGAACTTTTCAATGGCTTGACTGATCTCTTAGGTCACTTTTCCGAACGGCGTGTTCTCGGGAACTATCATCTCCAGTGTGATCCACAACGGCGATATCAAGAAAACTGCTGAATGGCAGCTTTTACTCGGAGCAATAGCCCTGCCTGGCGTCCTCGTCGGCGCTTGGCTCTGCAACCGACTGGGGAGAAGGAACACAGTATGCGACACACGACCCGCATTCATTCAAGCTACTCATCCTCTACCCCTTAGATGATTTTAGGATTCTCTGGATACCTAGTATTTGTAGGTCTTTCTCCTGCTTCCAGCTCATCATTTGCTACAATTGGCACGCCAGGGCCTCATAATTGGTCTCGCATACGATGAAATAACCAACATTATACCCCTCTTTGTAATTTTGTGGGTGTCATCAAAAATCCTTCACTAAACTGAGCTGAGAGCTGTCTTGTAGCTATGGGCTGATGCAGTCGTTTGGTAACCTGGTGCGTCACACTCGAGGTCGATCAGCTTAGTTGTTCAGATGATTACAGGGACCTGGTGATATGCTGGGCCTGACGAGTTCTGAGTAAGTCCATCGCAACTCCCTATTTACCTTGAAACTTAGGGGCCACACCACAGGTCGTATGCAACGCCCGTTCGGTATGACGCTAGCTCACGTTGAGTTGAAGAGACTCAATTTGATTGTCAGTGGAACATGTTACGGCCTTTCTGCAGCCATCGGTAAAACTGGCGCTGCTATTGGGACCCAAGCATTCACGCCTATCCAGAATAATCTCGGAAAACGGTACGAGTACTTCCCTTGCTTTCTTCCTTCTTGCTGATCCAGACAATGAAGTTGGACTTTCATCATCGCCGCTATTTGCGGTATTGCTGGAATCCTTGTGACGTACTTCTTCGTGCCTGACATGACTGGCATTGACCTCGCGGACGAAGATGCCAAATTCATGAAGTACCTCGCAGAGAATGGTTGGCAGGGCGAGGTTGGAGAGGACGACGACAAGGCGTTGATTGGCACATATGCCAGTGAAGACGCCCTGGAGGAGAAGATCTGATATGAGATTAGTATATGTCCGTCACGATGGTATCATATGGGTAGTTCACCGTCTTTGCAACTTTTGAATTGGCGGAGGGTTGAAGATCACGTGCGCACTGAGGATTTCGCGTATTACTACTATTAGACTACAGTTGCTCAGCACATCTTGCCTTGAATGTCTGCTCAGGTTCTCCGGCAGTGCGCGTCTCGTCGTTCTTTGTGTTTCTCGTGTCTACAGTTTCGCCGCGCCAAAGCATACTACTCGCAATCAGCTCTCGCTTTTTCCTCCACCA

>*PiPT7_* gm1.7522_g

ATTTCTTTGGCGGCTACGCATGACGGAACCACCAAGCTACAAGAAGAACTCCATGAAGGCTGTGCGAATCCCATACTGGCTTATCGTCAAGCGTTACTGGAAAGGCCTCTTCGGACTTTCGCTAGCTTGGTGAGTTGGGAAGTACTTCGCGGATGGATACCACGTTAAACGTGCGCAAAACACAGGTTCATCTATGATTTCATCACGTACGTACGCTTGCTTAGTCTTCCAATGGTGGTTCTGAGATGTGCATACTCAGGTACCCGGTAAGATAGCCATCGCCACTACCAGCGCAATCCGCTCATAATGTTTGCAGTTCGGGATCTACTCTTCCACGATCACCAACAAGTGAGATGTCTCTGACACTCGACAGGTAATTGCATTACTGAGGCCATGACTCTTACAGCGTCACTGGAGGCAGCACCTCTTTGACAGTCGTGTTCGGCTGGAGTGTTGTTATTAAGTCAGTTGCCATTCTTCTTGCCGTATATTGTAAAACTCACGCATCTGGCCCAAGCTTGTTCTACATACCAGGTTCGTTTTTAACCAATTTCACCCTTGCGTGTATTCAAGGATATCGCAGGGACCGTCATCGGTGCCTTCGCGGTCGACTACCTGGGCCCAAAAACAACGATGGTAGTTTTGCATGTTTCGTGACAGTAACGACAACCCTGACGGTTGTTTTTAGATCTCTGGTCTACTTGCGCAATCTGTCATGGGTTTCATTATGAGTGGTCTATACACTCACCTCACCAAAAACATCGCTGCGTTCGCAGTAAGTTACTAGAACGTACAATCGCTATTCTCAATTGAGCCTTAAGCACAGGTCGTCTATGGGATCTTCTTGAGCTTTGGAGAGTTTGGTATGTCACTCGTCGAAATCGTCAAAACTCTTTCTTATAGGTGCACAGGACCCGGGAACTGTCTTGGTGTACTTGCTTCGAAGAGCGGACCCACTGCCGTTCGTGGCCAATACTACGGTGTCGCTGCGGCCGTCGGAAAGATTGGCGCATTCATTGGTACATGGTGTACGTGGAACATGCTGGTCTTTGCCATCATTAACTCATGTGATCATTGGTGAAATAGTCTTGCCTAACATCATCGCTGGTAACTAGTTACCATAACCGAATAAACGCCTGAGCTCACAGACTTTACGCAGACTTTGGCGGCGCCGATACGGCGAAGGGTAACACAGGCCCCTTCTGGATTGGAAGTGGCCTTGCCATCCTCAGTGCCCTGCTCATATTCTTCCTTGTTAGGCCTCTGTCGCATGACGGAATGAAGGAGGAAGACGAGAAGGTATGTTCCTGGTCAGAATCCATCCGGATGTGCCTGCTCTGACGTCGGATGACGCAGTTCCGTTTGTATCTGGAGGAGCACGGGTTTGACACATCATTGATGGGTATCCCTGACTCGGAAGTTTCGTCCACGATCTCCGAGGAAGAGAAAGTAGCTGCTTAATGAATGGGACATTGGGTCCTACAGCAAGATATCACACTTTTGACGATGATGAAAGATTACAAGCAATGTTGGGTCTTAGTAAATACGATGATCTTGTGCTTCTGGCTCTGCGTACCTGTAGCAAGTACTTGGCTAATGAATTTGTACGATGTTCTTGTTTGGCTCGCGTGAACAAACGCAGTGTTTCAGTTTGCTTTGGT

**Notes S2** Amino acid sequences of the *P. involutus* *ATCC 200175* putative PTs.

>PiPT1_169850
MASNADRLSDRASYEKNLTPANLNERRRAALQEIDNAPFSWFHVKVCLVAGVGFFTDAYD
IFAINIAVTMLGYVYGKNAALTPWQSTGLKAATPVGNLVGQLVFGWLADVLGRKRMYGIE
LMIMIIATFAQAVSGEAPAISIVGVLIVWRFLMGIGIGGDYPLSAIITSEFAATRSRGRL
MTAVFAAQGWGQLAAALVALVVVSAYKEAILAGPFPSALPIDHAWRLLIGLGCVPGVVAL
YFRLTIPETPRFTMDVERNVAQASQDIENVLTIGKHVEAEDAVVERAKAPRATRADFVAY
FSKWENSKVLVGTAYSWFALDIAFYGLGLNSSIILSAIKFGGSTAKKGTTLNVYESLHNI
CIGNIILSVAGLIPGYWVSFLFIDTWGRKPIQLMGFTALTILFVIMGFAYDKLVATTSGT
NVFVFLYCLTNFFQNFGPNTTTFIVPGEAFPTRYRSTAHGISAAAGKLGAVVAQLGFGQL
VNIGGTNKFVKHIMEIFAFFMLTGIFSTLLIPETKQKSLEDLSNEDQAEYITDS*

>PiPT2_18830
MTFLTSFSAPRHVNEAQPAYDLFIINPVATMLQYRLYGGQSLPNNLQGVLKASANIGSVI
GQFLFGYCADAFGRKAIYGKELMLIILATILCISCPTNLLSPDGSLIYLSIFRILLGVGV
GGDYPMSASITSDRAVLRKRGTLLAYIFANQGWGSLVGSLATIIVLACYKHVMNDEGETS
KVDGVWRIVVGLSLIPAFGTLYQRLTLPESVRFEESRKLTAGELDDLKAKNDVEITVSSK
NSNEGHVKEPLEKKAHFKEFVEYFSEWRHAKILIGTCTCWFLLDVAFYGINLNQNVVLQQ
IGYAGKTGTAWEKLFKVSTGNIIITALGFVPGYYVTVLTIEKLGRKYIQIQGFLMTSLFL
GILAGKFHTFDNASFIVCFSFLQFFFNFGANATTYCYPAEVFPTKYRASAHGLSAACGKA
GAIISALGFNTLTAKIGTPAVLWIFFGCSIAGAGFTLLLPEVAGRDPDAILAEELRER*

>PiPT3_165085
MSAEKDVPTLEAEQTTDPRNVSVKPRETNLIIAPNNLAKRESLSAWLTICAAGFGLISDG
YQNNLMTMSNVIFKTLYPKDYTSAVSTRVSNALLVGAVIGQIFVGLICDRAGRKVALVAT
TLFIVLGSTLGTAAHGANGSAQGLFWFLTFARGITGIGVGGEYPASSTSASEAANEKTQK
NRGPVFIMVTNFVLSFGGPLAVSVFLIALSAAGENHLQTVWRVCFGVGILLPLTVLVFRL
RMLSSKLYRKGAIKRDVPYGLVIKRYWRPLIGTCGAWFLYDFVTFPNGVFSGTIISSVIH
NGDIKKTAEWQLLLGAIALPGVLVGAWLCNRLGRRNTMILGFSGYLVFGLIIGLAYDEIT
NIIPLFVIFYGLMQSFGNLGPGDMLGLTSSESYATPVRGTCYGLSAAIGKTGAAIGTQAF
TPIQNNLGKRWTFIIAAICGIAGILVTYFFVPDMTGIDLADEDAKFMKYLAENGWQGEVG
EDDDKALIGTYASEDALEEKI*

>PiPT4_141142
MASHIEEKPISSAETGTPSAPFRSELQAYERDAGDRPPYVLTYPEIKLLGIAGVGFFLDA
YDLFIINPVATMLQYRLYGGQSLPNNLQGVLKASANIGSVIGQFLFGYCADAFGRKAIYG
KELMLIILATILCISCPTNLLSPDGSLIYLSIFRILLGVGVGGDYPMSASITSDRAVLRK
RGTLLAYIFANQGWGSLVGSLATIIVLACYK*

>PiPT5_163069
MPGLKDLSSVSHIFACGTALFSDGYANSVIGAVLERIYGSDVMTANNHSKAFTSVTFAGT
VVGMLIFGWVSDKIGRKFGMISATGIVALFILSAASAGANNSVAGMLAMLAACRFLIGIG
IGAGYPCGTVSASEQSEEGAIAKNAQHRFGNNHIRAVWRLSLGLGVVPALAVLLWRLRMT
EPLSYKDSMKSARIPYWLVVKRYWKGLFAPSPSWFTYDFISYTYISSTITNNVTGNNTSS
IVVFGWSVVVNLFYMSSTIIGAFLVDYWGPKATMIAGLLAQAIVYGIFLSFGEFDLGGDK
TAKGNTGPFLVGSGLAILSALVVFFLVRPLSYDGMKEEDVKFRMHLEGPGFDTSLMGVPD
SEVSSTISEEEKAAQTA*

>PiPT6_158250
MSAMASHIGEKPNSPGETGTSSAPLRSGLQAYERNVGDRPPFILTYSEIKLLGIAGVVWR
IVVGLSLIPAFGTLYQRLTLPESVRFEESRKLATGELDDLKAKNDVEIIVSGENSNEGDL
KERTEKKAHFKEFVEYFSEWRHAKILIGTCTCWFLLDVAFYGINLNQNVVLQQIGYAGDT
ATAWEQIFKVSTGNIIITALGFVPGYYVTVLTIEKLGRKYIQIQGFLMTALFCEPASYFF
AFSHSNTRPVLLQLWSQCFVQLTVSTPTDRTTYCYPAEVFPTKYRASAHGLSAACGKAGA
IISALGFNTLTAKIGTPAVLWIFFGCCIAGAGFTLILPEVAGRDPDAILAEELRER*

>PiPT7_12951
MTEPPSYKKNSMKAVRIPYWLIVKRYWKGLFGLSLAWFIYDFITYPFGIYSSTITNNVTG
GSTSLTVVFGWSVVINLFYIPGTVIGAFAVDYLGPKTTMISGLLAQSVMGFIMSGLYTHL
TKNIAAFAVVYGIFLSFGEFGPGNCLGVLASKSGPTAVRGQYYGVAAAVGKIGAFIGTWF
LPNIIADFGGADTAKGNTGPFWIGSGLAILSALLIFFLVRPLSHDGMKEEDEKFRLYLEE
HGFDTSLMGIPDSEVSSTISEEEKVAA*

**Notes S3** Analysis of Amino acid sequence similarities of the *P. involutus* *ATCC 200175* putative PTs PiPT3, PiPT5 and PiPT7 with other organisms (protein BLAST).

**PiPT3:**

| **Description** | **Max Score** | **Total Score** | **Query Cover** | **E value** | **Percentage Identity** | **Accession** |
| --- | --- | --- | --- | --- | --- | --- |
| hypothetical protein PAXINDRAFT_165085 [Paxillus involutus ATCC 200175] | 1013 | 1013 | 100% | 0 | 100.00% | KIJ21649.1 |
| hypothetical protein PAXRUDRAFT_823203 [Paxillus rubicundulus Ve08.2h10] | 944 | 944 | 100% | 0 | 93.61% | KIK99036.1 |
| hypothetical protein HYDPIDRAFT_44726 [Hydnomerulius pinastri MD-312] | 869 | 869 | 100% | 0 | 87.65% | KIJ57885.1 |
| hypothetical protein JAAARDRAFT_386367 [Jaapia argillacea MUCL 33604] | 768 | 768 | 87% | 0 | 86.79% | KDQ63656.1 |
| hypothetical protein PHLGIDRAFT_99480 [Phlebiopsis gigantea 11061_1 CR5-6] | 734 | 734 | 84% | 0 | 85.11% | KIP11646.1 |
| MFS Git1p-like glycerophosphoinositol permease [Dichomitus squalens] | 748 | 748 | 87% | 0 | 84.04% | TBU61970.1 |
| MFS Git1p-like glycerophosphoinositol permease [Dichomitus squalens LYAD-421 SS1] | 747 | 747 | 87% | 0 | 84.04% | XP_007359839.1 |
| MFS general substrate transporter [Heliocybe sulcata] | 776 | 776 | 92% | 0 | 83.01% | TFK57191.1 |
| hypothetical protein CERSUDRAFT_54709 [Gelatoporia subvermispora B] | 740 | 740 | 90% | 0 | 82.82% | EMD34864.1 |
| MFS Git1p-like glycerophosphoinositol permease [Dichomitus squalens] | 731 | 731 | 87% | 0 | 82.70% | TBU34588.1 |
| hypothetical protein BN946_scf184726.g4 [Trametes cinnabarina] | 723 | 723 | 88% | 0 | 82.33% | CDO78229.1 |
| MFS general substrate transporter [Coniophora puteana RWD-64-598 SS2] | 798 | 798 | 98% | 0 | 81.78% | XP_007762776.1 |
| MFS Git1p-like glycerophosphoinositol permease [Trametes coccinea BRFM310] | 718 | 718 | 90% | 0 | 81.76% | OSC99525.1 |
| MFS general substrate transporter [Rhizopogon vinicolor AM-OR11-026] | 803 | 803 | 99% | 0 | 81.71% | OAX44721.1 |
| MFS Git1p-like glycerophosphoinositol permease [Trametes versicolor FP-101664 SS1] | 749 | 749 | 90% | 0 | 81.54% | XP_008032905.1 |
| MFS Git1p-like glycerophosphoinositol permease [Schizopora paradoxa] | 760 | 760 | 92% | 0 | 81.51% | KLO20255.1 |
| hypothetical protein CY34DRAFT_24989 [Suillus luteus UH-Slu-Lm8-n1] | 821 | 821 | 99% | 0 | 81.33% | KIK40010.1 |
| MFS general substrate transporter [Suillus brevipes Sb2] | 819 | 819 | 99% | 0 | 81.33% | KAF3877737.1 |
| MFS Git1p-related glycerophosphoinositol permease [Laccaria bicolor S238N-H82] | 687 | 687 | 89% | 0 | 81.03% | XP_001873845.1 |
| MFS Git1p-like glycerophosphoinositol permease [Dichomitus squalens] | 777 | 777 | 95% | 0 | 80.88% | TBU46102.1 |
| hypothetical protein D9619_003260 [Psilocybe cf. subviscida] | 741 | 741 | 90% | 0 | 80.71% | KAF5313240.1 |
| hypothetical protein HYPSUDRAFT_61451 [Hypholoma sublateritium FD-334 SS-4] | 750 | 750 | 90% | 0 | 80.62% | KJA29420.1 |
| MFS Git1p-like glycerophosphoinositol permease [Stereum hirsutum FP-91666 SS1] | 785 | 785 | 97% | 0 | 80.53% | XP_007307286.1 |
| hypothetical protein TRAPUB_6420 [Trametes pubescens] | 759 | 759 | 93% | 0 | 80.47% | OJT03077.1 |
| hypothetical protein AZE42_00275 [Rhizopogon vesiculosus] | 797 | 797 | 99% | 0 | 80.40% | OJA10783.1 |
| putative inorganic phosphate transporter [Heterobasidion irregulare TC 32-1] | 782 | 782 | 97% | 0 | 80.37% | XP_009540336.1 |
| MFS Git1p-like glycerophosphoinositol permease [Gloeophyllum trabeum ATCC 11539] | 784 | 784 | 97% | 0 | 80.25% | XP_007861071.1 |
| MFS Git1p-like glycerophosphoinositol permease [Pluteus cervinus] | 771 | 771 | 97% | 0 | 80.25% | TFK74737.1 |
| MFS Git1p-like glycerophosphoinositol permease [Sanghuangporus baumii] | 786 | 786 | 97% | 0 | 80.24% | OCB85689.1 |
| MFS Git1p-like glycerophosphoinositol permease [Dendrothele bispora CBS 962.96] | 760 | 760 | 94% | 0 | 80.21% | THV04837.1 |
| hypothetical protein SERLADRAFT_472685 [Serpula lacrymans var. lacrymans S7.9] | 812 | 812 | 99% | 0 | 80.20% | XP_007320734.1 |
| MFS Git1p-like glycerophosphoinositol permease [Polyporus brumalis] | 735 | 735 | 90% | 0 | 80.13% | RDX50399.1 |
| hypothetical protein EVG20_g1951 [Dentipellis fragilis] | 802 | 802 | 98% | 0 | 79.96% | TFY71057.1 |
| MFS Git1p-related glycerophosphoinositol permease [Crucibulum laeve] | 715 | 715 | 92% | 0 | 79.91% | TFK41053.1 |
| putative glycerophosphoinositol permease [Moniliophthora roreri MCA 2997] | 721 | 721 | 92% | 0 | 79.62% | ESK98300.1 |
| hypothetical protein PILCRDRAFT_817340 [Piloderma croceum F 1598] | 786 | 786 | 98% | 0 | 79.56% | KIM85338.1 |
| MFS general substrate transporter [Ganoderma sinense ZZ0214-1] | 753 | 753 | 91% | 0 | 79.50% | PIL31631.1 |
| putative MFS general substrate transporter [Moniliophthora roreri] | 719 | 719 | 92% | 0 | 79.41% | KTB42812.1 |
| hypothetical protein PLICRDRAFT_396161 [Plicaturopsis crispa FD-325 SS-3] | 765 | 765 | 96% | 0 | 79.30% | KII93547.1 |
| MFS Git1p-like glycerophosphoinositol permease [Fibularhizoctonia sp. CBS 109695] | 777 | 777 | 97% | 0 | 79.22% | KZP31225.1 |
| MFS Git1p-like glycerophosphoinositol permease [Dentipellis sp. KUC8613] | 785 | 785 | 99% | 0 | 79.12% | KAA1468145.1 |
| hypothetical protein WOLCODRAFT_77494 [Wolfiporia cocos MD-104 SS10] | 751 | 751 | 95% | 0 | 79.08% | PCH44779.1 |
| hypothetical protein K443DRAFT_88209 [Laccaria amethystina LaAM-08-1] | 670 | 670 | 89% | 0 | 79.06% | KIK06890.1 |
| hypothetical protein EW145_g1533 [Phellinidium pouzarii] | 704 | 704 | 90% | 0 | 78.82% | THH10148.1 |
| predicted protein [Fibroporia radiculosa] | 716 | 716 | 92% | 0 | 78.71% | XP_012184381.1 |
| MFS general substrate transporter [Rickenella mellea] | 787 | 787 | 97% | 0 | 78.60% | TDL28370.1 |
| Major glycerophosphoinositol permease GIT3 (GroPCho permease GIT3) (Glycerophosphodiester transporter GIT3) [Ganoderma boninense] | 759 | 759 | 96% | 0 | 78.47% | VWO94238.1 |
| uncharacterized protein SCHCODRAFT_65057 [Schizophyllum commune H4-8] | 724 | 724 | 89% | 0 | 78.44% | XP_003037582.1 |
| MFS Git1p-like glycerophosphoinositol permease [Sistotremastrum niveocremeum HHB9708] | 758 | 758 | 96% | 0 | 78.41% | KZS96082.1 |
| hypothetical protein EW026_g2534 [Phlebia centrifuga] | 773 | 773 | 99% | 0 | 78.31% | THG99889.1 |
| MFS Git1p-like glycerophosphoinositol permease [Pyrrhoderma noxium] | 769 | 769 | 97% | 0 | 78.28% | PAV22202.1 |
| MFS Git1p-like glycerophosphoinositol permease [Punctularia strigosozonata HHB-11173 SS5] | 766 | 766 | 100% | 0 | 78.24% | XP_007379105.1 |
| major facilitator superfamily domain-containing protein [Auriculariopsis ampla] | 722 | 722 | 89% | 0 | 78.17% | TRM65680.1 |
| permease [Steccherinum ochraceum] | 778 | 778 | 99% | 0 | 77.89% | TCD69968.1 |
| MFS general substrate transporter [Neolentinus lepideus HHB14362 ss-1] | 773 | 773 | 99% | 0 | 77.80% | KZT30475.1 |
| hypothetical protein GALMADRAFT_233721 [Galerina marginata CBS 339.88] | 709 | 709 | 96% | 0 | 77.73% | KDR85098.1 |
| uncharacterized protein PHACADRAFT_258580 [Phanerochaete carnosa HHB-10118-sp] | 732 | 732 | 96% | 0 | 77.59% | XP_007397294.1 |
| MFS Git1p-related glycerophosphoinositol permease [Exidia glandulosa HHB12029] | 704 | 704 | 89% | 0 | 77.46% | KZV93275.1 |
| hypothetical protein EWM64_g3532 [Hericium alpestre] | 766 | 766 | 98% | 0 | 77.42% | TFY80480.1 |
| hypothetical protein EUX98_g622 [Antrodiella citrinella] | 762 | 762 | 98% | 0 | 77.39% | THH33504.1 |
| hypothetical protein M422DRAFT_210104 [Sphaerobolus stellatus SS14] | 766 | 766 | 98% | 0 | 77.33% | KIJ39867.1 |
| hypothetical protein POSPLADRAFT_1139705 [Postia placenta MAD-698-R-SB12] | 729 | 729 | 92% | 0 | 77.30% | XP_024340377.1 |
| MFS Git1p-like glycerophosphoinositol permease [Lentinus tigrinus ALCF2SS1-6] | 773 | 773 | 99% | 0 | 77.00% | RPD64897.1 |
| hypothetical protein GYMLUDRAFT_90105 [Gymnopus luxurians FD-317 M1] | 717 | 717 | 92% | 0 | 76.94% | KIK71113.1 |
| MFS Git1p-related glycerophosphoinositol permease [Auricularia subglabra TFB-10046 SS5] | 711 | 711 | 89% | 0 | 76.67% | EJD41536.1 |
| MFS Git1p glycerophosphoinositol permease [Fomitopsis pinicola FP-58527 SS1] | 717 | 717 | 93% | 0 | 76.50% | EPS95589.1 |
| MFS Git1p-like glycerophosphoinositol permease [Polyporus arcularius HHB13444] | 758 | 758 | 100% | 0 | 76.25% | TFK89640.1 |
| hypothetical protein CVT26_002154 [Gymnopilus dilepis] | 705 | 705 | 92% | 0 | 76.09% | PPQ64447.1 |
| MFS Git1p-related glycerophosphoinositol permease [Laetiporus sulphureus 93-53] | 719 | 719 | 94% | 0 | 76.06% | KZT00653.1 |
| hypothetical protein BOTBODRAFT_490216 [Botryobasidium botryosum FD-172 SS1] | 715 | 715 | 93% | 0 | 75.48% | KDQ10576.1 |
| MFS Git1p-like glycerophosphoinositol permease [Obba rivulosa] | 718 | 718 | 95% | 0 | 75.16% | OCH87787.1 |

**PiPT5:**

| **Description** | **Max Score** | **Total Score** | **Query Cover** | **E value** | **Percentage Identity** | **Accession** |
| --- | --- | --- | --- | --- | --- | --- |
| hypothetical protein PAXINDRAFT_163069 [Paxillus involutus ATCC 200175] | 764 | 764 | 100% | 0 | 100.00% | KIJ15663.1 |

**PiPT7:**

| **Description** | **Max Score** | **Total Score** | **Query Cover** | **E value** | **Percentage Identity** | **Accession** |
| --- | --- | --- | --- | --- | --- | --- |
| hypothetical protein PAXINDRAFT_12951 [Paxillus involutus ATCC 200175] | 536 | 536 | 100% | 0 | 100.00% | KIJ14301.1 |
| hypothetical protein PAXRUDRAFT_823601 [Paxillus rubicundulus Ve08.2h10] | 511 | 511 | 100% | 4.00E-179 | 93.63% | KIK98691.1 |
| hypothetical protein HYDPIDRAFT_113854 [Hydnomerulius pinastri MD-312] | 472 | 472 | 98% | 9.00E-164 | 84.85% | KIJ62762.1 |
| hypothetical protein M404DRAFT_991412 [Pisolithus tinctorius Marx 270] | 419 | 419 | 98% | 1.00E-142 | 76.89% | KIO14651.1 |
| MFS general substrate transporter [Suillus brevipes Sb2] | 421 | 421 | 98% | 2.00E-143 | 76.14% | KAF3881672.1 |
| hypothetical protein PISMIDRAFT_672928 [Pisolithus microcarpus 441] | 419 | 419 | 98% | 8.00E-143 | 76.14% | KIK28745.1 |
| hypothetical protein CY34DRAFT_12860 [Suillus luteus UH-Slu-Lm8-n1] | 416 | 416 | 98% | 2.00E-141 | 75.76% | KIK41723.1 |
